# A gender perspective on the global migration of scholars

**DOI:** 10.1073/pnas.2214664120

**Published:** 2023-02-27

**Authors:** Xinyi Zhao, Aliakbar Akbaritabar, Ridhi Kashyap, Emilio Zagheni

**Affiliations:** ^a^Department of Digital and Computational Demography, Max Planck Institute for Demographic Research, Rostock 18057, Mecklenburg-Vorpommern, Germany; ^b^Leverhulme Centre for Demographic Science, Department of Sociology, University of Oxford, Oxford OX1 1JD, UK; ^c^Nuffield College, University of Oxford, Oxford OX1 1NF, UK

**Keywords:** global migration of scholars, gender gap, bibliometric data, science of science, feminization of global migration

## Abstract

Although considerable progress toward gender equality in science has been made in recent decades, female researchers continue to face significant barriers in the academic labor market. International mobility has been increasingly recognized as a strategy for scientists to expand their professional networks, and that could help narrow the gender gap in academic careers. Using bibliometric data on over 33 million Scopus publications, we provide a global and dynamic view of gendered patterns of transnational scholarly mobility, as measured by volume, distance, diversity, and distribution, from 1998 to 2017. We find that, while female researchers continued to be underrepresented among internationally mobile researchers and migrate over shorter distances, this gender gap was narrowing at a faster rate than the gender gap in the population of general active researchers. Globally, the origin and destination countries of both female and male mobile researchers became increasingly diversified, which suggests that scholarly migration has become less skewed and more globalized. However, the range of origin and destination countries continued to be narrower for women than for men. While the United States remained the leading academic destination worldwide, the shares of both female and male scholarly inflows to that country declined from around 25% to 20% over the study period, partially due to the growing relevance of China. This study offers a cross-national measurement of gender inequality in global scholarly migration that is essential for promoting gender-equitable science policies and for monitoring the impact of such interventions.

## Significance

Within a globalizing scientific system, international migration is increasingly recognized as a strategy for scientists to advance their careers. The migration literature more broadly has suggested a process of feminization, with an increasing share of women among all international migrants. With respect to the migration of scholars, however, whether male and female scholars participate equally in transnational mobility and how these patterns have shifted over time from a global perspective are not known. Using bibliometric data that cover the past two decades, we show that, while female researchers continued to be underrepresented among internationally mobile researchers, and migrated over shorter distances, this gender gap has been narrowing at a faster rate than the gender gap in the population of general researchers.

Over the past 50 y, women have made enormous strides in scientific research, including in the fields of science, technology, engineering, and mathematics (STEM) (1, 2). Nonetheless, women continue to face a number of barriers to participation, recognition, and progression in the scientific arena (3–5). In the current era of globalization, international mobility is increasingly recognized as a key strategy for scientists seeking to participate in global scientific networks and collaborations and to advance their careers (6, 7). However, less attention has been paid to gender differences in international scholarly migration, especially on a global basis (3, 5, 8, 9). Our study considers the interplay between the globalization of scientific knowledge, the internationalization of academia, and gender inequalities in the academic labor market (10–12), with the aim of providing substantive support for policies that advance gender equality in academia.

While the population of female scientists and scholars has more than doubled since 1993 and a wide array of programs promoting gender equality in academia have been launched, gender disparities persist in nearly all facets of academia and sciences (8, 13). In 2016, women researchers held 41% of academic positions across the 28 countries of the European Union (EU-28). However, in many European countries, including in the Netherlands and Germany, women held fewer than one in five senior academic positions (13). Women are also underrepresented as researchers in Asian countries such as Japan, where they account for only approximately one in four full-time faculty members (14). Female researchers in the Global South are relatively “invisible” compared to those in the Global North (15), and their representation among researchers in Guinea (6%), Ethiopia (7.6%), and Mali (10.6%) shows more alarming gender disparities (16). While it is clear that the sciences and academia continue to be dominated by males at the global scale, there is also substantial variation in levels of gender inequality across countries. Unfortunately, unified and comprehensive statistics suitable for making cross-national comparisons of gender disparities in the sciences do not exist (17–19), let alone statistics on gender disparities in global brain circulation. The first goal of our study is to document cross-national trends in a systematic way.

Existing research that has considered the gender dimension in international scholarly migration has mainly focused on either emigrants from an origin country perspective (20, 21) or on immigrants from a destination country perspective (11, 22, 23). Although some of these studies have discussed scholarly mobility involving several countries, most have paid little attention to their separate roles as receiving and sending countries, mainly owing to a lack of relevant data (21). Within the global migration system, more broadly, the increasing interconnectedness and integration of countries around the world have led to a more distinct pattern of international migration, where migrant populations have been coming from an increasingly diverse range of countries but have been moving to a shrinking number of prime destination countries (24). A similar pattern has also been observed among highly skilled migrants (25). Looking specifically at the population of academic scientists, movements from the Global South to the Global North, and from Asia to English-speaking countries like the United States, the United Kingdom, and Canada have been the long-established paths (26–28). In recent decades, also as a result of numerous programs aimed at attracting overseas researchers, and of changes in socioeconomic and geopolitical conditions, non–English-speaking western countries, like Switzerland, Germany, and France (29, 30), as well as some Asian countries, such as China and Singapore (31–33), have become increasingly attractive for international researchers. However, the extent to which destinations are diversifying, or established asymmetric and skewed patterns among researchers are changing, has not been assessed in depth.

Emerging evidence has shown that men and women do not respond to the regional pull and push factors with the same intensity when making migration decisions (20). Despite the increasing average migration distances for mobile researchers (34, 35), women are more likely to concentrate in the largest urban centers. Also, wherever they reside, women are less likely than men to relocate (36, 37). Generally, female researchers are less geographically mobile than their male counterparts (38). From the perspective of reconciliation of career and family, married academic men are more likely than academic women to relocate to small communities where there are fewer academic positions available and are less likely than women to choose positions in large metropolitan areas (37). By contrast, women academics are more likely than their male counterparts to make job shifts within the same locality instead of pursuing cross-border mobility. Given these considerations in the literature, we hypothesize that female researchers generally migrate shorter distances and have a lower diversification level in both scholarly immigration and emigration, compared to their male counterparts.

Existing research highlights geographic constraints on women’s careers in academia (39), and evidence is accumulating that large differences in labor market conditions and in women’s rights between the origin and the destination countries have led to larger migration flows of highly skilled females to specific destinations (25, 40, 41). Thus, gender disparities in specific bilateral migration corridors may vary substantially, contributing to distinct migration trajectories and distributions of female and male researchers. More broadly, it has been argued that social inequalities are produced and reproduced within the globalization of the science system (42–44). In particular, gender plays a significant role in shaping international academic mobility patterns (42, 45–48), and underrepresentation of female migrant researchers has been observed, in various degrees, in country-specific analyses (38, 42, 49, 50). Due to the lack of time series of comparative and gender-disaggregated data on migration of scholars, we do not have a clear picture of gender inequalities in global patterns of migration of scholars. However, based on the literature, we hypothesize that, while women are generally underrepresented in academia and among internationally mobile researchers, there is also substantial heterogeneity in levels of gender inequality across countries.

The migration literature, more broadly, has highlighted growing feminization of migration, indicating an increasing share of women among all migrants and the tendency of women to migrate more independently of men (51–53). The idea of feminization of migration has grown in importance in the new age of international migration and globalization, with the doubling of female migrants during the period 1960 to 2015, and with relatively equal shares of women and men in the migrant population (51, 52, 54). The feminization of international migration not only relates to the increasing figure of female migrants but also to the fact that women increasingly migrate independently, in search of jobs, instead of depending on marriage and families (55, 56). Research has also shown increasing proportions of well-educated women from the Global South (or low- and middle-income countries) concentrating in more economically developed countries in the Global North (20, 40, 57). For example, the comparison between the education- and gender-specific worldwide migration in 1990 and that in 2000 indicated an increasing participation of high-educated women in South-to-North emigration (20). However, the question of whether the feminization process has also emerged for global transnational scholarly mobility, and exhibits similar patterns, has received limited attention. In light of these trends suggestive of feminization of international migration, and also because of the emergence of an increasing number of programs for supporting female researchers in academic mobility to Global North destinations (58), we hypothesize that the underrepresentation of women among internationally mobile scholars has been decreasing over time and that an increasing number of female scholars from the Global South have been concentrating in more economically developed countries in the Global North.

To fill these and related gaps in our understanding of gender inequalities in scholarly migration, and to test our hypotheses, we use data from Scopus on over 33 million publications. Our data and methods enable us to estimate international mobility of researchers from around the world, and by gender, during the 1998 to 2017 period. We aim to assess how gender inequality among mobile academic scientists varies across countries and over time on a global scale and how it affected the demographic composition of the scientific workforce across the origin and the destination countries.

## Results

### Over the Study Period, Women Scientists Were Less Internationally Mobile than Men, but the Gap Shrank Considerably.

Worldwide, the number of researchers who have published in Scopus-indexed outlets has increased considerably during the past decades. Our analyses show that the number of female published researchers in the 2013 to 2017 period was nearly three times as large as in the early 1998 to 2002 period, rising from approximately 0.7 to 1.7 million. There was also a substantial increase in the number of male published researchers: Over the same period, the number doubled from 1.5 to 3 million. We also find a considerably increasing migration intensity of female researchers, in terms of both absolute numbers and the proportion of all female published researchers. The number of female mobile researchers nearly tripled over this period, rising from 29,000 (4.3% of all published female researchers) in 1998 to 2002, to 79,000 (4.6%) in 2013 to 2017. By contrast, over the same period, the population of male mobile researchers grew more slowly, roughly doubling in absolute count from 92,000 (6% of all published male researchers) to 167,000 (5.6%). This trend suggests growing feminization of international scholarly migration, in line with our hypothesis. The migration intensity of specific countries is shown in *SI Appendix*, Table S3.

**Fig. 1.**
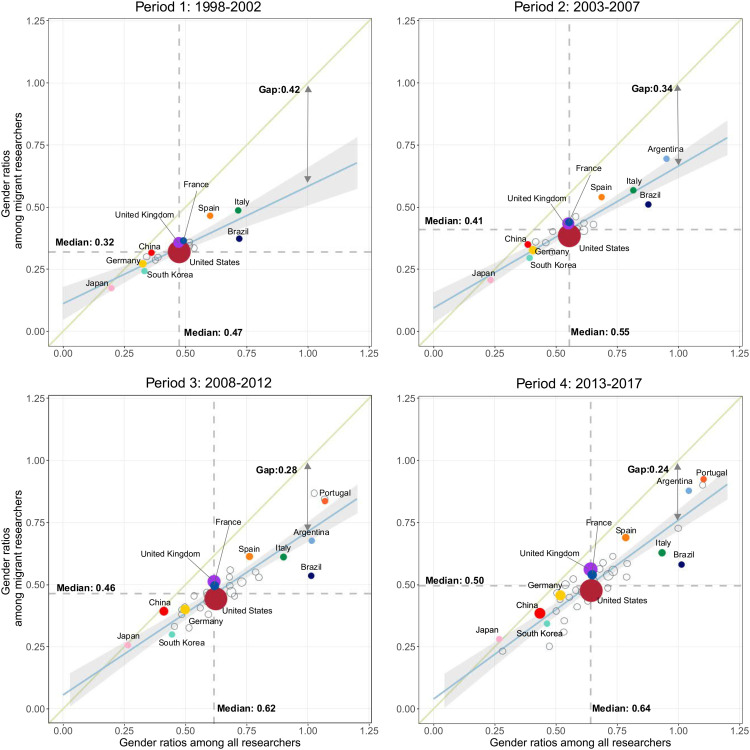
Gender ratios among all published researchers (X-axis) and migrant researchers (Y-axis). In the subfigure for each period, only the countries with over 500 female mobile researchers are shown, as including the countries with small populations of mobile researchers may give rise to bias in the ratio measurements. The size of each country’s circle is proportional to the number of female researchers who migrated from and to this country. Notably, to increase readability, the countries with no more than 2,000 female migrant researchers are set to the minimum size. The vertical and horizontal dashed lines indicate the median gender ratios of all published researchers and of mobile researchers in each period. The 45° line in each subfigure is used to help compare the gender ratios of these two categories, with another double-arrowed line underlining the distance between it and the fitted regression line at the X value of one. This helps us to track the convergence tendency of female representation in the group of mobile researchers versus that in the total researcher population and how it changed over the four time periods.

To better understand the gendered patterns of global scholarly migration over time, we computed a female-to-male gender ratio that measures the gender gaps among all published researchers (x-axis) and migrant researchers (y-axis), as shown in Fig. 1. The migrant researchers depicted here include both emigrants from, and immigrants to, each country. (Gender ratios separated by incoming and outgoing researchers are shown in *SI Appendix*, Fig. S1.) Fig. 1 shows that the overall median gender ratio in both groups increased over the study period, from 0.47 to 0.64 among all published researchers and from 0.32 to 0.5 among the subgroup of mobile researchers. The gap between the fitted regression line and the 45° line shank gradually, declining from 0.42 to 0.24. This pattern indicates that the increase in the share of females among migrant researchers outpaced the increase of females among all researchers. In other words, the gender gap in scholarly mobility was decreasing at a faster pace than the overall gender gap among researchers. Further examination by field of specialty shows that, although the level of the gender gap among mobile researchers varies by fields of specialty, the trend of decreasing gender gap among mobile scholars is present across fields over this period (Standardization by fields of specialty section in *SI Appendix*).

Gender parity, or even higher representation of female researchers (female-to-male ratio of one and above), were achieved in only a small fraction of countries, most noticeably in Portugal and Serbia, where the gender ratios for mobile researchers also approached one. While the values of the two gender ratios were correlated (i.e., in most countries, if female researchers were less represented among all published researchers, they were also less represented among migrant researchers), the values of the gender ratios among migrant researchers were typically smaller than the values of the gender ratios among all researchers. This indicates that female researchers were less internationally mobile than male researchers in almost all of the shown countries. A similar pattern can also be observed in both migration inflows and outflows (*SI Appendix*, Fig. S1).

In addition, Fig. 1 also shows that three clusters of countries have emerged over time. In the first cluster of countries, located mainly in the bottom-left quadrant, there were marked gender disparities over time (e.g., Saudi Arabia, Pakistan, and South Korea). Female scholars in these countries were underrepresented in the population of scholars and in the subgroup of mobile researchers, and their situation did not improve substantially over the four periods. In the second cluster comprising a large share of countries, the proportions of female researchers among mobile scholars and among all active scholars have remained close to the global median values over time. This group included the largest and more established science systems (e.g., the United States, the United Kingdom, Canada, and Germany). The third cluster comprised the group of countries that achieved gender equality at the level of the population of researchers and that also had a relatively high female-to-male ratio among mobile researchers (e.g., the countries in the top right parts of the figure panels, such as Serbia, Argentina, and Portugal).

Looking at countries based on the World Bank classification of income groups (i.e., high-income, upper-middle-income, lower-middle-income, and low-income economies) shows a similarly increasing trend in the female-to-male ratios of both researchers and mobile researchers across all income groups, except among low-income countries where the gender ratio declined during the 2013 to 2017 period (*SI Appendix*, Fig. S7). Across all periods, high-income and upper-middle income countries showed smaller gender gaps in global scholarly migration, and among researchers more generally, than low-income countries. Despite this, low-income countries had the smallest difference between gender ratios among migrant researchers and gender ratios among all researchers. This suggests that, in low-income countries, female researchers may be a highly selected group, given the overall larger gender inequalities in science in these settings. The greater propensity to migrate among female researchers may also reflect gender norms and poor job opportunities in low-income countries that act as push factors for more skilled women to migrate (51, 59).

### Female Mobile Researchers, on Average, Migrated Shorter Distances and Concentrated in a Relatively Narrow Range of Destination Countries, Compared to Male Mobile Researchers.

Both male and female mobile researchers migrated increasingly longer distances (*SI Appendix*, Fig. S2) which is in accordance with previous findings for general patterns of global migration (35), despite a slight drop in the migration distance of female researchers over the period 2013 to 2017. Our hypothesis that male researchers tend to migrate longer distances than female researchers holds on a global scale. When disaggregated by country, there are a few exceptions such as China and South Korea (*SI Appendix*, Fig. S3). Female researchers migrating from China and South Korea, on average, moved longer distances than their male counterparts. This finding challenges some existing hypotheses that Asian women are more likely to migrate to neighboring East Asian countries and the Middle East (56).

To deepen our understanding of the characteristics of global scholarly migration by gender, we further investigate the spreads of migration outflows and inflows. These measurements can help to quantify the extent to which mobile researchers were dispersed across destination countries (emigration spread) and the extent to which they came from a diverse range of origin countries (immigration spread) (24, 35). (See the definition of migration spread measures in the *Measures of Migration Spreads by Gender in the Data, Methods, and Measurements section*.) The aim of gender-disaggregated analyses is to assess whether female and male outflows were spread equally across the destination and the origin countries.

When we look at emigration, we see that across the four periods, there were, respectively, 103, 119, 137, and 148 distinct origin countries for female researchers and 141, 149, 160, and 164 distinct origin countries for male researchers. Conversely, when we look at immigration, we observe that across the four periods, there were 105, 126, 140, and 147 countries that received female immigrant researchers and 144, 154, 168, and 173 countries that received male immigrant researchers. Overall, these numbers indicate that more countries were engaged in international academic circulation over time but also that male researchers originated from and moved to more countries than female researchers in each period, albeit with a narrowing of the gender gap across periods. While the numbers of distinct origin and destination countries were relatively balanced for female mobile researchers, the volume of academic destinations for male researchers increased faster than the range of origin countries.

**Fig. 2.**
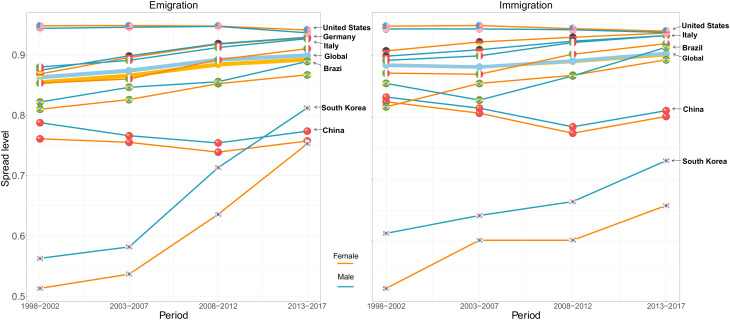
Scholarly emigration (*Left*) and immigration (*Right*) spreads, by gender and across four periods, for selected countries (labeled lines) and at the global level (solid, thicker line without a flagged circle).

Fig. 2 shows the emigration (*Left*) and immigration (*Right*) spreads of a selection of countries, with the global (country-weighted) levels of spread indicated by the solid line without a flag. (Further information on methods and analyses with different global measures is provided in *SI Appendix*.) The countries were selected as the representatives of the three clusters in Fig. 1, where gender disparities among mobile researchers were relatively large in South Korea and China, but small in Brazil and Italy. Germany and the United States were located in the largest cluster of countries with moderate values of gender ratios, close to the global median. These two countries are also among those that have well-developed science systems and are established academic magnets for global researchers. Additionally, the six countries are located across Asia, Europe, North America, and South America.

The solid lines without flags in Fig. 2 indicate that global migration spreads among both female and male researchers underwent a stable increasing trend along the two dimensions of emigration and immigration. By the fourth period, the values of the emigration and the immigration spread had converged to a similar level. However, the gender gap in global emigration spreads (blue solid line versus orange solid line) suggests that female mobile researchers were, overall, concentrated in a narrower range of destination countries across the four periods.

Increases in the dispersion of outflows and inflows are broadly observed in most countries, but to varying degrees. The most noticeable increase that can be seen in Fig. 2 occurred in South Korea, a country that lagged behind other countries in earlier periods and then experienced rapid growth in both immigration and emigration spreads. For China, by contrast, we observe a declining trend in the diversification of both outflows and inflows relative to the earlier periods. These trends indicate that, over time, the outflows of academic researchers of both genders from China have tended to concentrate in a narrower pool of destination countries, and the inflows have been more unevenly dispersed in a shrinking number of origin countries over time. The increasing intensity in the volume of scholarly migration to and from China, but with decreasing migration spreads, indicates strengthening scientific relationships between China and some specific countries. For example, the proportion of high-tech research in China that was conducted in collaboration with the United States increased continuously, from 4.6% in 2009 to 16.9% in 2019. (60). While the United States remained the country with the largest emigration and immigration spreads, throughout the four periods, it also saw a slight decrease in the migration spreads in the most recent period.

In most countries included in Fig. 2, the spread values were lower for female mobile researchers which indicates that there was less diversification in both origin and destination countries among female mobile researchers than among male mobile researchers. South Korea stood out as having a clear-cut gender gap in migration spreads: Compared to their female counterparts, male researchers leaving South Korea consistently migrated to a broader range of destinations, and male researchers entering South Korea also had higher levels of diversification in their origin countries. The rapid increase in the migration spreads of both female and male researchers did not help to narrow the gender gap in migration diversities. In contrast, in the United States and Germany, female mobile researchers had higher levels of immigration spread than male mobile researchers, which indicates that the female researchers who moved to these countries were more evenly dispersed in their origin countries than their male counterparts.

Furthermore, levels of emigration and immigration spreads among researchers across the Global South, defined as belonging to low- and lower-middle income countries, were higher (*SI Appendix*, Fig. S8). This suggests a type of pattern where researchers from high-income countries tended to concentrate in other high-income countries, in contrast to those from low- and lower-middle-income countries, who tended to migrate to a broader range of destinations. Moreover, low-income countries exhibited larger gender gaps in migration spreads in both directions. In other words, the difference between migration spreads between men and women were larger in this set of countries than in those at other income levels.

### Distribution of Bilateral Scholarly Migration Flows by Gender.

#### Favored destinations at the global level.

**Fig. 3.**
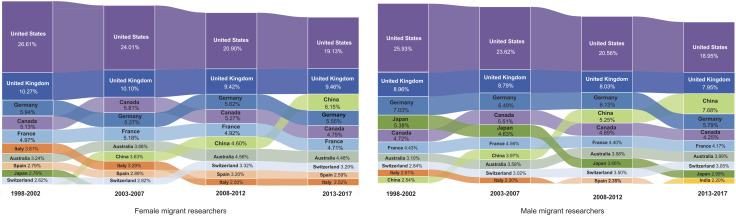
The 10 most preferred destination countries for female mobile researchers (left) and male mobile researchers (right). The labeled number for each country is the proportion of inflows each country received among all global migration flows by gender. The order of the 10 countries from the top to the bottom reflects the level of their attractiveness from the highest to the lowest.

The increases in emigration spreads indicate that mobile researchers became increasingly distributed across a more diverse range of destination countries. This trend has shifted the landscape of globally attractive destinations for research talent. Fig. 3 shows in more detail the dynamics of the 10 most preferred destination countries, together with the respective gender-disaggregated shares of inflows. The United States, the United Kingdom, and Germany were consistently attractive to large shares of female and male mobile scholars. For researchers of both genders, the United States had the largest incoming flows, receiving more than one-quarter of global mobile researchers in the first period, and nearly one-fifth of global mobile researchers in the latest period. In the most recent period, China replaced Germany as the third-most popular host country for mobile researchers of both genders. Following closely behind were Canada and France, which received comparable shares of mobile researchers. Japan, by contrast, has been losing its attractiveness for mobile researchers over time. In addition, Japan was an academic destination that was more favored by male researchers than by female researchers.

More distinct gender disparities can be observed among countries in the lower positions, among the top 10 destinations. The composition and the ranks of the countries ranking seven to 10 for male researchers showed greater variability over time, than the preferred destinations for female migrant scholars. For instance, India emerged recently as the 10th most preferred destination, accounting for more than 2% of male inflows, overtaking Spain in the third period, and Italy in the second period. By the fourth period, both Italy and Spain were no longer among the 10 most preferred destinations for male researchers. In comparison, Italy and Spain continued to receive larger shares of female inflows, in line with the observation in Fig. 1 that the female-to-male ratios of migrant researchers in the two countries were relatively high. Over time, the migration flows for researchers of both genders became more evenly distributed across the top 10 destinations and became less concentrated among the top three destinations. This finding is consistent with the long-term increases in emigration spreads, as shown in Fig. 2 and in *SI Appendix*, Fig. S4.

#### Favored destinations at the country level.

**Fig. 4.**
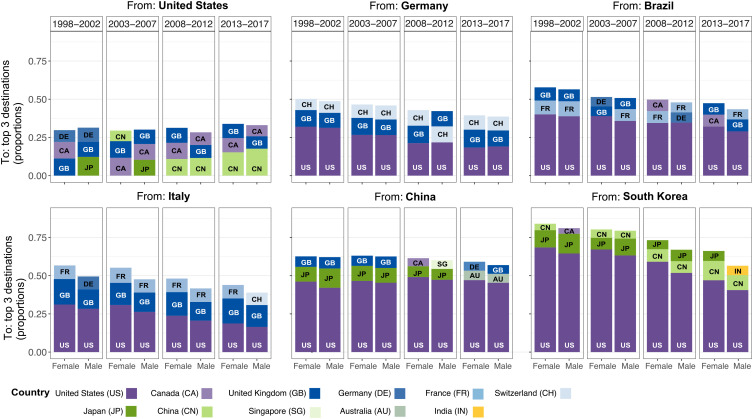
Top three destinations for mobile researchers by gender at the country level. Stacked bars are ordered based on the share of mobile researchers who migrated to each of the top three destination countries (indicated by different colors and labels).

Fig. 4 presents the three most preferred destinations for female and male mobile researchers from the six countries with different levels of diversification (shown in Fig. 2). The differences in the distribution of outflows provide a more detailed explanation of the varying patterns of the country-level migration spreads and migration paths. The outflows to the top three destinations across these countries ranged from a minimum level of concentration of 25% (see the case of the United States) to a high level of concentration of 75% (see the cases of South Korea in the two earliest time periods). In the case of the United States, the more balanced distribution of outflows to host countries corresponded to a much higher level of emigration spreads from the United States, as shown in Fig. 2. Meanwhile, scholarly emigration from South Korea was considerably skewed toward the United States, indicating the lower diversity of scholarly outflows.

Looking specifically at these countries, the United States attracted the largest share of mobile researchers from other countries and was especially favored by female researchers. This observation can help to account for the lower emigration spreads observed in these countries. In contrast, the spreads of the outflows of female and male researchers from the United States were more balanced across the top three emigration destinations. A notable change for the United States in the two most recent periods was the growing share of its outflows to China, which have helped China become one of the most popular destination countries from a global perspective (shown in Fig. 3).

The destinations of mobile researchers from each country differ by gender to varying degrees. For instance, while Japan is among the top three destinations for outgoing males from the United States in the first two periods, it was not so for females. Female mobile scholars from all selected origin countries, with the exception of the United States in the two earliest periods, were always more likely to concentrate in the top three destination countries with slightly larger proportions. This greater concentration of female scholars among the top three destinations was consistent with the generally lower emigration spreads among female scholars than among male scholars. While the largest shares of migration inflows were to the United States, the migrations of researchers of both genders tended to link the countries that had stronger cultural ties and close geographic proximity. For instance, Switzerland (as a German-speaking country) was among the top destinations for Germany’s outgoing scholars. Despite the United States leading the other countries with a large share of immigrant researchers from South Korea by a wide margin, China was also consistently preferred as a destination by migrants from South Korea and other Asian countries. This preference for destinations with cultural similarities and geographic proximity was consistent across the four periods.

## Discussion

Using bibliometric data on over 33 million publications, this study has provided a global view of transnational scholarly migration by gender over the 1998 to 2017 period. Our analysis revealed a gender gap in favor of males among all published researchers and an even larger gender gap among internationally mobile researchers. However, the rate of increase in female representation among global mobile researchers outpaced the rate of increase among all scholars, which suggests that female researchers have become more internationally mobile over the study period, in both absolute and relative terms. These trends indicate that broader patterns of increasing feminization of international migration have also occurred for global scholarly migration, a specific type of highly skilled mobility. Despite these increases, significant cross-national heterogeneity persisted, with some countries, such as Serbia, Argentina, and Portugal, having near gender parity (female-to-male ratio of one) among migrant researchers, while some countries, such as Japan and South Korea, having significant gender gaps in favor of men (around 0.25). Global talent hubs like the United States and Germany saw gender gaps around the global median levels (around 0.6).

In contrast to the skewed patterns of globalization in international migration, which indicate that migrants tended to move from an increasingly diverse range of origin countries to a shrinking pool of prime destination countries (24), our analysis showed that, for scholarly migration, there were simultaneous trends toward increasing migration distance and diversification of origin and destination countries among both male and female mobile researchers. The declines in transportation and communication costs, together with the strengthening of collaboration between national universities, likely contributed to greater levels of knowledge diffusion within a more balanced, globalizing science system. Despite this increasing diversification in the global scholarly migration system, women continued to migrate shorter distances, on average, and had lower emigration spreads, as they moved to a narrower range of destination countries than men. This gender gap in emigration spreads was more pronounced in countries that had large gender gaps in transnational scholarly mobility, such as South Korea.

The existing literature has pointed out that the global scientific system is largely shaped by highly resourced nations (61), with the United States being the primary destination of choice for researchers (9, 30, 49, 62, 63). Our results revealed a more nuanced picture, showing that the share of researchers moving to the United States steadily declined over time, by around 7% for both male and female inflows over 20 y, despite its unchanged position as the top destination. Additionally, female researchers were consistently more likely than their male counterparts to choose the United States as their academic destination, from both a global and a country-level perspective. Meanwhile, China has emerged as a prime destination with continuously increasing shares of female and male immigrant researchers, primarily from the United States and from neighboring Asian countries. Concurrently, a large number of PhD students and early-career researchers funded by the Chinese government to study abroad are required to return after finishing their studies, which also makes China an emerging destination for researchers who had originally moved from China (identified as Chinese returnee researchers) (64).

While our study has provided a comprehensive picture of the gendered pattern of global scholarly migration, it also has limitations that we would like to acknowledge. We considered researchers who are published scholars whose information was retrieved from the Scopus database. This is a database which is dominated by English language journals mostly situated in western countries (e.g., EU countries, the United States, and the United Kingdom). Despite this, approximately 22% of titles in Scopus are published in languages other than English (adding up to 40 local languages), and more than half of Scopus content originates from outside North America (65). In other words, Scopus has a relatively large coverage of documents in non-English language. In addition, internationally mobile scholars are more likely to publish at least part of their scholarship in English. Given the prominent role of English in science, we do not have reasons to believe that this bias would affect the key results of the article in a substantial way. However, it is important to remember that Scopus is not representative of all scholars, as non-English publications are underrepresented. Future research could explore avenues for combining different and complementary data sources in order to make these types of data more broadly representative. Another limitation is potential inaccuracies in the gender detection results for Chinese researchers. Compared to the detection results for other countries, our design process, which is consistent with state-of-the-art approaches, is less accurate in detecting genders from transliterated Chinese names. For the purposes of this study, we performed a sensitivity analysis by imputing missing gender (*SI Appendix*, *Imputation for missing gender*), which confirmed the robustness and reliability of the results that we present in the article. A promising direction for future research on gender detection includes applying our gender detection methods to names written in Mandarin, rather than to names transliterated in the Roman alphabet (2, 66). We also note that our study identifies scholars’ origins in terms of their country of academic origin (See the *Data*, *Methods* *and* *Measurements* section for more details), which may not necessarily be their country of nationality or ethnic origin.

Our findings describe the global gender imbalances in scholarly migration but do not assess the specific mechanisms or factors underlying the imbalances or the factors driving changes in the gender gap. The promising trend toward the closing of the gender gap suggests that the targeted scholarship and fellowship programs funded by governments, multilateral organizations, and private foundations have helped women advance their academic careers through relocation, at least to some extent (67, 68). However, other factors, such as relocation for family reasons, might have also pushed female researchers to migrate more frequently or be considered nonmovable and be excluded from the hiring pool (69). Thus, the relocation decisions of female scholars may not be attributable to their individual motivations for career advancement or research opportunities (41). Therefore, the autonomy and the freedom to engage in international migration should also be considered when examining the decision-making of female researchers or the negotiations of academic couples (70). This study represents an initial key step toward improving our understanding of global patterns over time, which is essential for promoting gender-equitable science policies and interventions. We encourage future research, both within specific country contexts and from a comparative perspective, to deepen our understanding of the factors that contribute to the aggregate-level patterns that we observed.

## Data, Methods, and Measurements

### Bibliometric Data on Global Publications from Scopus.

Our study relied on a large-scale bibliometric dataset consisting of more than 33 million Scopus article and review publications and on an exhaustive dataset covering 10 million published researchers worldwide between 1996 and 2020, which have been disambiguated using Scopus author identification numbers (71). While Scopus covers a longer time period, we used the period window starting in 1996 due to license limitations and the quality of the metadata. After dealing with missing countries, the cleaned and preprocessed bibliometric information allowed us to map the geographic locations of the published researchers and to extract the authorship by linking each author’s affiliation and publication (9, 63). These processing steps laid the foundation for identifying mobile researchers and their transnational trajectories and for analyzing patterns of global scholarly mobility over time.

### Migration Flows and Researcher Population.

Transnational academic migrants were identified based on whether the authors had ever been affiliated with universities or institutes in a country other than their country of origin through their publications (2, 9, 72). To detect the potential migration events, the residence country(ies) in each year was assigned to each author according to his or her most frequent (mode) country(ies) during the publishing year and that of the most recent years of publishing, if needed. We assumed that an author migrated when his or her country of affiliation changed (9, 73). Given that authors may not publish every year, some host countries could be missed during the gaps between the publication years. These authors did not contribute to the researcher population in those nonpublication years, which could have led to an underestimation of the researcher population. To prevent this, we implemented a two-year padding (vicinity) to fill the gaps in the years between publications and to estimate the annual researcher population. We provide more details in *SI Appendix*. To reduce the effects of yearly fluctuations and to compare the migration trends over time, we considered four time periods (1998 to 2002, 2003 to 2007, 2008 to 2012, and 2013 to 2017) and grouped the migration events according to the migration year. Notably, we excluded the years 1996, 1997, 2018, and 2019 in the time periods due to the application of the two-year padding method in the population estimation.

### Gender Detection from First Names.

The only form of Scopus metadata that could be used to infer an author’s gender was the author’s first name (74). Before making name-to-gender inferences, we handled the issues related to problematic first names in the Scopus data, including inconsistent names for a single author, combined names, and unavailable names. We have listed these issues and the corresponding solutions in *SI Appendix*.

We then elaborated a sequential, three-step process to infer the gender identities of researchers from their first names by using 1) a large dictionary of names and genders, i.e., a worldwide gender-name dictionary (WGND) that includes 6.2 million names from 182 different countries (75); 2) a gender detection tool Demographicx based on a deep learning Bidirectional Encoder Representations from Transformers (BERT) embedding model with subword tokenization (76, 77); and 3) an application programming interface (API) called genderize.io. The genders of the majority of names were identified through WGND, and the remaining first names without genders were further processed, first using Demographicx and then using genderize.io.

To ensure the reliability of our gender detection process, we validated the developed method against two established databases of first names and genders (19, 78), and we also compared the detection performance of our method and another gender detection method (79) (*SI Appendix*). We found that 31.55% of the authors were female, and 57.72% of the authors were male, while the genders of the remaining 10.73% of authors were labeled as “unknown.” To test the robustness of our results given the missing gender, we assigned the gender of female or male to the researchers missing specific gender information using Multiple imputation by chained equations (MICE) (more detail in *SI Appendix*, *Imputation for missing gender*). The results after gender imputation display a consistent pattern with the original results before gender imputation.

### Measures of Migration Spread by Gender.

spread To explore the diversification of the origin and the destination countries over our study period, we used a migration spread measure (24) that showed how dispersed or concentrated migration trajectories were across all observed bilateral migration corridors in terms of both the origin and the destination countries. Specifically, the emigration spread (*ES*_*t*_^*i*^) defined in (**1**) is used to measure the extent to which bilateral migration flows ($M_{t}^{\mathrm{ij}}$) from any given country *i* are diverse in the destination countries during a specific period of time *t*.

[1] $$ES_{t}^{i}=1-\sum_{j=1}^{n_{t}}{\left(\frac{M_{t}^{ij}}{EM_{t}^{i}}\right)}^{2},$$

*n*_*t*_ is the number of countries involved in the global scholarly migration during the period *t*. $M_{t}^{\mathrm{ij}}$ indicates the size of the emigration flows from country *i* to country *j*, and *EM*_*t*_^*i*^ measures the overall volume of the emigration flows from country *i* by adding up all bilateral emigration flows from country *i*. Similarly, we calculated the immigration spreads (*IS*_*t*_^*i*^) to measure the diversity level of the inflows $M_{t}^{\mathrm{ji}}$ to country *i*, relative to all the immigration flows to country *i* (*IM*_*t*_^*i*^). At the global level, we used two methods to calculate the migration spread, shown in *SI Appendix*. We disaggregate the migration spreads by gender to examine whether the female and the male researchers came from and moved to an equally diverse range of countries.

## Supplementary Material

Appendix 01 (PDF)

## Data Availability

We used the bibliometric data through the German Competence Centre for Bibliometrics (Kompetenzzentrum Bibliometrie, grant number 16WIK2101A, https://bibliometrie.info/) and the access they granted to the Max Planck Digital Library. Sharing the individual-level original raw data with third parties is against the data usage agreement. We curated an anonymized, aggregate version of the data, that removes the commercial value of the data but maintains its scientific value. Scripts and data which allow for replication of our analysis are publicly accessible on GitHub under https://github.com/zxy919781142/A-gender-perspective-on-the-global-migration-of-scholars.
