# Supplementary material for: A gender perspective on the global migration of scholars
Source: Proc Natl Acad Sci U S A. 2023 Feb 27;120(10):e2214664120. doi: 10.1073/pnas.2214664120 (PMC10013742; doi:10.1073/pnas.2214664120)
Supplement: Supplementary file 1 — Appendix 01 (PDF) [file pnas.2214664120.sapp.pdf]

# Supplementary Information for

## A gender perspective on the global migration of scholars

**Xinyi Zhao, Aliakbar Akbaritabar, Ridhi Kashyap and Emilio Zagheni**

**Corresponding Author: Xinyi Zhao.**
**E-mail: zhao@demogr.mpg.de**

**This PDF file includes:**

Supplementary text
Figs. S1 to S12
Tables S1 to S3
SI References

## Supporting Information Text

## Methods

**Migration events.** For any author $A$, we can obtain a sequential collection of publications, composed of the corresponding publishing years and affiliation countries. The publication collection is defined as:

$$\text{Publications}(A) = \{(T_1, C_1), (T_2, C_2), ..., (T_i, C_i), ..., (T_n, C_n)\} \quad [1]$$

Here, $T_i$ and $C_i$ represent the publishing year and mode residence country(ies) of the $i$th publication and $n$ is the number of all publications of author $A$.

To determine the moving year between $T_i$ and $T_{i+1}$ in the case of migration when $C_i$ is different from $C_{i+1}$, we first assume the middle year between $T_i$ and $T_{i+1}$ is $T_{mid}$, which is calculated by the ceiling method (ceiling is a function that maps any real number to the least integer greater than or equal to this number). For example, $T_{mid}$ is 2003 when $T_i$ and $T_{i+1}$ are 2001 and 2004. We then consider the following circumstances to determine the migration events of author $A$:

(a) If $T_{mid}$ is within the two-year padding threshold of $T_{i+1}$, that is, $(T_{i+1} - T_{mid}) \leqslant 2$, we take $T_{mid}$ as the moving year $T_{move}$ in which the author moves from $C_i$ to $C_{i+1}$. For example, when $T_i$ and $T_{i+1}$ are 2001 and 2004, we consider the movement that happened in 2003.

(b) If $T_{mid}$ is outside the two-year padding threshold of $T_{i+1}$, that is, $(T_{i+1} - T_{mid}) > 2$. In this case, there is a large period gap between the two publishing years. Thus, we apply the two-year padding (vicinity) method by taking $(T_{i+1} - 2)$ as the moving year $T_{move}$. For example, when $T_i$ and $T_{i+1}$ are 1999 and 2007, the year 2005 is determined to be the moving year. This is based on the assumption that each publication signals the output of work that has happened in the past two years (to account for the publication delays in different disciplines) (1).

In this case, we can obtain this movement event Migration $= \{T_{move}, C_{origin}(\text{i.e.,}C_i), C_{destination}(\text{i.e.,} C_{i+1})\}$ for author $A$. Similarly, the author who has at least one movement during her or his publishing career can be identified as a migrant researcher, and the respective movements can be extracted from the publication information.

**Researcher population by year.** Given the two sequential publication instances $(T_i, C_i)$ and $(T_{i+1}, C_{i+1})$ of author $A$, when $T_i$ and $T_{i+1}$ are not consecutive years, we fill the gap to estimate the researcher population by considering the two-year padding of $(T_i, C_i)$ and $(T_{i+1}, C_{i+1})$. Table S1 lists all possible situations that can be used to fill the gap between $T_i$ and $T_{i+1}$.

Similarly, the filling instances before $T_i$ and those after $T_i$ can be determined by considering the extracted information from adjacent publications. The filling instances are then used to estimate the researcher population for countries in each year. For example, in case 2, author $A$ can be considered as an active author in $C_i$ during the years $T_i$ and $T_i + 1$. In addition, the author is assumed to be involved in the researcher population of $C_{i+1}$ during the years $T_{i+1} - 1$ and $T_{i+1}$.

**Problematic first names for gender identification and implemented solutions.** In the following, we discuss the issues related to problematic names that happened when we attempted to determine the genders of the authors' first names, and the strategies we adopted to deal with them:

(a) *Inconsistent names*: One author ID (i.e., the unique identification number assigned by Scopus to differentiate and disambiguate authors (2)) may have multiple name variations (e.g., the records of the same author ID may have the names of Christine and Christina, as illustrative examples).
**Solution**: The most frequent (mode) name was extracted to assign to this author in order to ensure that each author ID has only one single first name.

(b) *Combined names*: Some first names include special characters or are combined with a middle name (e.g. David A., H. Okti, and Yan Ying).
**Solution**: If the first name string contains the initials and names (e.g. David A., H. Okti), the non-initials were kept as their first names (e.g., David, Okti); if the string contains multiple non-initials (e.g., Yan Ying), the first non-initial was taken as the first name (Yan).

(c) *Unavailable names*: Some first names may only have initials (e.g. A., H.)
**Solution**: The genders of these authors were categorized as "unknown."

**Validation of gender detection.** The first established database we used for validating our gender detection process is a name-gender dictionary of 16,921 female first names, 17,740 male first names, and 9,907 unisex first names in 68 countries worldwide (3). The second dataset we used was a manually calibrated dataset of 4,558 authors (1,447 female authors, 2,980 male authors and 131 authors without identified genders) extracted from top sociology journals (4). We employed the indicators of *precision* and *recall* rate to separately judge the detection accuracy for female and male first names in different countries. The *precision rate* of female names quantified the proportion of the correctly identified female first names among all the first names we identified as female, while the *recall rate* quantified the proportion of the correctly identified female first names among all the female first names in the name-gender dictionary. The two indicators were also applied to the male first names.

For the validation of the first dataset, our gender detection process worked well in almost all countries. The evaluation results for a portion of the countries or regions are shown in Table S2. In general, the *precision rate* and the *recall rate* were over 90%, which can be seen as proof of the overall high degree of accuracy of our gender detection method. China was an exception, mainly because in the name-gender dictionary, a large share of Chinese first names were unisex in the transliterated Roman alphabet.

For the second database, the *precision rate* and the *recall rate* for the female authors' first-names were 0.97 and 0.80, respectively; while the *precision rate* and *recall rate* for the male authors' first-names were 0.98 and 0.86, respectively.

To compare our gender detection method with methods used by other research, we applied another name-gender database generated by a gender disambiguation algorithm (5) in detecting the genders of the researchers in our dataset. This reference database included the genders of 1,609,107 distinct US first authors from the Web of Science. The comparison of the results of the two methods shows that the genders of 89.27% of global researchers are identified consistently (Specifically, the genders of 94.23% of migrant researchers and 88.69% of non-moving researchers, respectively, were identified consistently).

**Global migration spread.** Similar to the country-level migration spread, the global emigration spread ($ES_t^{Global}$, Eq. (2)) and the global immigration spread ($IS_t^{Global}$, Eq. (3)) measure the extent to which the global migration flows were dispersed across all destination and origin countries during the period $t$.

$$ES_t^{Global} = 1 - \sum_{i=1}^{n_t} (\frac{EM_t^i}{M_t})^2 \quad [2]$$

$$IS_t^{Global} = 1 - \sum_{i=1}^{n_t} (\frac{IM_t^i}{M_t})^2 \quad [3]$$

Additionally, given the hypothesis that the migration spreads varied across countries over time, different countries have played different roles in the global migration spread. We also measured the weighted average of the country-specific emigration spread ($ES_t^{Weighted}$, defined in Eq. (4)) to represent the overall level of emigration spread during the period $t$. Accordingly, the global weighted average of the country-specific immigration spread ($IS_t^{Weighted}$, defined in Eq. (5)) can be calculated.

$$ES_t^{Weighted} = \sum_{i=1}^{n_t} ES_t^i \cdot \frac{EM_t^i}{M_t} \quad [4]$$

$$IS_t^{Weighted} = \sum_{i=1}^{n_t} IS_t^i \cdot \frac{IM_t^i}{M_t} \quad [5]$$

**Gender differences in migration distributions.** To a given destination country $i$, the average gender difference among migration inflows at a specific period of time $t$ is measured using Eq. (6),

$$Diff\_I_{i,t} = E(|\frac{IM_{f,t}^i}{M_{f,t}} - \frac{IM_{m,t}^i}{M_{m,t}}|/(\frac{IM_{f,t}^i}{M_{f,t}} + \frac{IM_{m,t}^i}{M_{m,t}})) * 100 \quad [6]$$

$IM_{f,t}^i$ and $IM_{m,t}^i$ indicate the immigration flows of female and male researchers to country $i$ from all possible origin countries during the period $t$. $M_{f,t}$ and $M_{m,t}$ are the number of all migration flows of female and male researchers during the period $t$, respectively. The ratio of the gender difference $|\frac{IM_{f,t}^i}{M_{f,t}} - \frac{IM_{m,t}^i}{M_{m,t}}|$ to the sum $(\frac{IM_{f,t}^i}{M_{f,t}} + \frac{IM_{m,t}^i}{M_{m,t}})$ reflects the *relative gender difference* along each migration path to country $i$. Considering the potential disturbance of extreme values measured from the single-gender dominant migration paths, we used the trimmed mean of the middle 70% of the *relative gender differences*. That means that the top 15% and the bottom 15% of the *relative gender differences* will be ignored, and not involved in this measurement. Finally, the trimmed average value $Diff\_I_{i,t}$ indicates the overall gender difference in immigration distribution of country $i$. If the female and male researchers immigrating to country $i$ share the same distribution of migration paths, $Diff\_I_{i,t}$ will be zero; otherwise, if the two groups follow totally different migration distributions, $Diff\_I_{i,t}$ will reach the maximum value, i.e., one. Similarly, $EM_{f,t}^i$ and $EM_{m,t}^i$ indicate the emigration flows of female and male researchers, respectively, from country $i$ to all possible destination countries during the period $t$. Accordingly, $Diff\_E_{i,t}$, the overall gender difference in the distribution of migration outflows from country $i$ at time $t$ is calculated as below, ranging from zero to one:

$$Diff\_E_{i,t} = E(|\frac{EM_{f,t}^i}{M_{f,t}} - \frac{EM_{m,t}^i}{M_{m,t}}|/(\frac{EM_{f,t}^i}{M_{f,t}} + \frac{EM_{m,t}^i}{M_{m,t}})) * 100 \quad [7]$$

## Further analysis and results

**Migration intensity of mobile researchers by gender.** The dataset we processed for our analysis on gender disparities in global scholarly migration involved 180, 191, 197, and 202 countries during the periods 1998–2002, 2003–2007, 2008–2012, and 2013–2017, respectively. To make the results more comparable, and to capture the main flows and dynamics of global scholarly migration over time, we estimated the migration intensity of mobile researchers by gender which includes both the absolute number of female (male) mobile researchers and their proportions relative to all female (male) researchers (relative number). Table S3 shows the results of the 18 major countries, which were the origin and also the destination countries with the largest 50 migration flows in any of the four periods. And these migration flows across the 18 countries accounted for 94.97%, 93.57%, 88.69%, and 80.88% of all scholarly migration flows over the four periods, respectively. The Table also indicates that global scholarly migration mainly occurred across a small fraction of countries, which led to the skweness of migration flows.

It is evident that the share of scholars and the size of the science system were much larger in some countries than others (e.g., see the case of the USA, which has hosted around one-third of the share of worldwide scholars in all time periods). The countries with the larger and more established science systems greatly influenced the observed migration trends.

**Gender disparities in transnational emigration and immigration .** The gender ratios varied among the outgoing and the incoming researchers, and thus contributed differently to the country-level gender disparities. Meanwhile, the overall gender ratio among migrants may obscure the divergent outcomes for transnational scholarly migrations in both directions. Fig. S1 shows the gender ratios among the outflows and the inflows separately to help us gain a better understanding of how the gendered patterns of scholarly mobility varied across the four time periods.

Generally, the countries appearing in all four figure panels experienced stable and slow growth in the female-to-male ratios in both directions. In addition, the countries emerging in the sub-plots of recent periods (especially period three and period four) that had sufficient numbers of female migrant researchers were more likely to display identifiable patterns of gender ratios far from the median values. These countries either had significant gender disparities (such as Pakistan and Saudi Arabia) or relatively balanced gender relationships (such as Portugal and Serbia). Taken as a whole, the gender patterns of scholarly inflows and outflows across most major countries were consistent over time

As outliers in the overall gender patterns of the scholarly inflows and outflows, several countries stood out as having a skewed gender ratio in one direction. One example is Brazil. In the most recent period of 2013–2017, around seven female researchers were leaving Brazil for every 10 male researchers who were emigrating. However, the number of female researchers moving to Brazil was nearly half that of male researchers. This indicates that the level of gender balance was higher among the researchers leaving Brazil than among the researchers moving to Brazil.

**Migration distance by gender.** We measured the migration distances based on the geographic distance between the capital cities of the origin and destination countries. As the Fig. S2 shows, there is a gap between the average distance of male and female researchers that is consistent in all time periods and males migrate longer distances. The average migration distances for both female and male researchers kept increasing until 2012. During the most recent period 2013-2017, the average migration distance for male researchers still increased but at a slower rate, while female researchers on average tended to migrate shorter distance compared to the previous period. Accordingly, the gender difference regarding the average migration distance became wider.

We also compared the average migration distances among female and male researchers across six selected countries in Fig. S3, which are also the countries shown in Fig. 2 in our main text. Migrant researchers across Asian countries like China and South Korea usually migrated longer distances compared to researchers in European countries such as Germany. The distribution of prime academic destinations plays an important role in determining migration distances. That is, the preferred destination countries are clustered in North America and Western Europe, far away from the East Asian countries. Therefore, we attached more attention to the destinations of scholarly migration by gender to dig into the globalization process and compare gender differences. The distribution of top destination countries by migrant researchers is shown in Fig. 3 (global level) and Fig. 4 (country level) in our main text.

**Global-level migration spread by gender.** Using the two measures described in *Methods: Global migration spread*, Fig. S4 shows that on a global scale, immigration and emigration spreads kept increasing over time, except for a slight decline in the overall (unweighted, lines with squares) emigration spreads and the country-weighted (lines with triangles) immigration spreads during the second period (2003–2007).

The overall (unweighted) migration spreads indicate that mobile researchers of both genders tended to migrate from a relatively small number of countries to a more diverse array of destination countries, especially in the earlier periods. By comparison, in the female migration pattern, there was a larger difference between the emigration and the immigration spreads, which indicates that there was less balance between the outbound and inbound flows among female researchers. That implies that overall, female researchers from a smaller group of countries were able to move internationally, and they were more likely to disperse across a broader range of destination countries.

Compared to the overall global-level migration spreads without weighting, the country-weighted average spread measures indicate that there was less diversification of the average migration spreads with smaller spread values, after incorporating weights that accounted for the size of migration flows across different countries. And it shows a more distinct gender pattern, especially among outflows where female mobile researchers were less diverse in their destination countries, which also corresponds to the country-level emigration spreads shown in Fig. 2, in the main text. The weighted values, in essence, give more importance

to the major migration countries, which accounted for the largest shares of international migration flows. This indicates that the scholarly inflows and outflows of the major migration countries were less diverse than the overall global scholarly flows, which is in line with the literature showing that a large share of migrant researchers moved from and to only a small number of countries (6–8). More interestingly, the figure of country-weighted migration spreads showing more diversification in scholarly inflows relative to scholarly outflows contrasted the figure of the overall migration spreads, especially during the earlier periods. The more diverse range of origin countries from the perspective of specific countries tended to overlap when aggregating, while the pool of destination countries of global outflows was less concentrated. More recently, the emigration and immigration spreads obtained from both methods gradually converged to similar values, which indicates that similar patterns of the diversification of scholarly inflows and outflows were occurring in more countries.

Strikingly, the simultaneously increasing emigration and immigration spreads among mobile researchers can be compared to a migration pattern among the general population by Czaika, and de Haas (9). Their analysis revealed that the immigration pattern of the general population has become more skewed and concentrated, with average migrants appearing to be concentrated in a smaller pool of fewer selected destination countries (declining emigration spreads), but to come from a larger pool of origin countries (increasing immigration spreads). However, our results show that among scholarly migrants, the pools of both the sending countries and the receiving countries have tended to become more diverse over time. Indeed, the migration patterns of scholars do not have to follow those of the general population. In the past few decades, technological shifts have led to increasing demand and competition for high-skilled workers among a growing number of countries. This trend has facilitated an increase in the diversity of academic destinations for high-skilled migrants, especially for researchers, and has led to more frequent global exchanges.

**Gender differences in migration distributions.** The gender differences in migration distributions point to the possible gender differences in the support provided for or the barriers to leaving a specific country, and also to the benefits or the costs of moving to a particular destination. We calculated the average gender differences among migration inflows and outflows using the methods in *Methods: Gender difference in migration distributions.*

Fig. S5 compared the gender differences in the immigration distribution (top half in four panels) and the emigration distribution (bottom half) of mobile researchers across different countries over time. Generally, the gender gaps along the inflow paths of researchers were larger than that along the outflow paths, especially for the first two periods. In particular, the countries such as Russia and Japan consistently had larger average gender differences among immigrants, staying near the top of the ranking. This indicates that for these countries, the trajectories of female and male immigration were less overlapped. By contrast, countries like the US, the UK, and France ranked last in terms of the gender gaps in the inflow distributions, with female and male mobile researchers following similar migration paths. Regarding the emigration, there were significant differences in the outward trajectories of female and male mobile researchers emigrating from the USA. The gendered distribution of scholarly inflows and outflows, combined with the levels of migration spreads, provide more information about the gender disparities in the national science systems under the current socio-spatial configurations. These insights are critical for understanding the future trends of high-skilled migration by gender.

**Gender disparities among preferred destinations.** To investigate how gender differences in preferences for destinations of scholarly migration varied by country over time, Fig. S6 shows the gender ratios of the shares of scholarly flows to each destination (Y-axis) from a given country (X-axis) over the four periods. The value in each cell can be calculated by Eq. (8). Taking the migration from the US ($i$) to China ($j$) as an example, in the first period, the proportion of female outgoing flows from the US to China among all female emigration flows from the US was 1.1 times the proportion of male flows from the US to China among all male emigration flows from the USA. This indicates China can be considered a destination for migrations from the US that was preferred slightly more by females than by males. In the latest period, the gender ratio of scholarly mobility along the same path became 0.87, making China a destination that was favored by male mobile researchers from the US. The cells labelled zero indicates there were no detected results for such routes among female researchers in that period.

$$Gender\_OD_{ij} = \frac{EM^{ij}_{f,t}}{EM^{i}_{f,t}} / \frac{EM^{ij}_{m,t}}{EM^{i}_{m,t}} \qquad [8]$$

The results highlight that the gendered patterns of scholarly migration tended to be more even and diverse over time, with a considerable share of extremely gendered migration flows turning into slightly single-gender-dominated or gender-balanced flows. It can be observed that overall, the cell colors were more neutral as the cell values approached one. For example, the migration flows from Germany to Italy, which tended to be dominated by female researchers in the earlier periods, became more gender-balanced in the latest period. Nevertheless, some countries continued to attract predominantly female or predominantly male mobile researchers. Italy and Spain were more popular among female researchers throughout the periods, as indicated by the warmer colors in the rows for Italy and Spain. Conversely, the migration flows of researchers to Japan, South Korea, and most Asian countries were consistently dominated by males, regardless of their origins (as indicated by the colder colors in the corresponding rows for these countries). In contrast to the countries that were preferred mainly by either male or female researchers, the US and the UK, as the largest receiving countries and also established scientific destinations for mobile researchers (6, 7), had relatively gender-balanced patterns of scholarly migration throughout the study periods.

## Aggregated analysis on income groups of countries

The aggregated income-group level analysis on gender patterns of global scholarly migration can help us further to examine if the closing gender gaps is consistent in both the Global North and Global South. Here we used the groups of countries by income from the World Bank (low-income economies, lower-middle-income economies, upper-middle-income economies, and high-income economies) (10). In our dataset, there were 28, 49, 51, and 68 countries in the groups of low-income economies, lower-middle-income economies, upper-middle-income economies, and high-income economies, respectively. We consider those in the group of low- and lower-middle-income economies as being in the Global South.

**Gender ratios among migrant researchers versus all researchers.** Overall, the Global North countries which include high-income countries and upper-middle-income countries achieved higher level of gender parity in global scholarly migration, shown in Fig. S7. By contrast, low-income countries saw the largest gender disparities among mobile researchers while lower-middle income countries continued to show the most under-representation of female researchers in the group of all published researchers.

Generally, the trend of gender ratios over the period 1998-2017 indicated an overall increasing representation of female researchers in both populations of researchers, except for the group of low-income countries with a declining trend of gender ratios in the most recent period. Despite this, low-income countries had the smallest difference between the gender ratios among migrant researchers and the gender ratios among all researchers. This suggests that in low-income countries, female researchers may be a highly selected group, given the overall larger gender inequalities in academia in these settings. It also may reflect the feminization of migration in Global South where gender norms and poor job opportunities may act as push factors for more skilled women to migrate (11, 12). For other groups of countries, especially the high income countries which have remained the dominant academic hubs accommodating a large part of researchers all over the world, an increase in the female representation of migrant researchers at faster pace echoes the findings in our main text that globally, female researchers have been more internationally mobile than ever before.

**Emigration and immigration spreads.** The increasing diversification of origin and destination countries among both male and female mobile researchers on the global level can also be observed in different income groups, shown in Fig. S8, especially the emigration spreads which indicates migrant researchers of both genders have been more widely dispersed in the destination countries. However, both emigration and immigration spreads among researchers across the Global South were at a higher level than those in the upper middle- and high-income countries. It echoes the effect of "rich-get-richer" where researchers from high-income countries tend to concentrate in other high-income countries, in contrast to those from low- and lower-middle-income countries, who tend to migrate to a broader range of destinations.

Despite the gender trend of higher diversification across destinations for researchers from the Global South, male mobile researchers from the Global South are still more diverse in a relatively broad range of destination countries, in line with our previous findings. Low-income countries exhibited larger gender gaps in the migration spreads in both directions, which tended to be wider in the most recent period.

## Robustness analysis

**Standardization by fields of specialty.** Here we used a category of six research fields: Agricultural Sciences, Engineering and Technology, Humanities, Medical and Health Sciences, Natural Sciences, and Social Sciences, which are a mapping of OECD categories (13) to the All Science Journal Classification Codes (ASJC) from Scopus. The mapping is done by the German Competence Centre for Bibliometrics. Only researchers with over 70% of their publications in a given field were assigned to that field of specialty to favor the field with the most proportion in the case of scholars with multidisciplinary publications. We first looked at the female-to-male gender ratios among both whole population of researchers and migrant researchers in the six fields of specialty at different time periods, shown in Fig. S9. When breaking it down by field, Social Sciences features more balanced gender ratios among all researchers, followed by the Medical and Health Sciences. However, among migrant researchers, the gender ratios in Medical and Health Sciences surpassed that in the Social Sciences, which indicates women studying Medical and Health Sciences are more likely to engage in global academic migration. By contrast, across both whole population of researchers or migrant researchers, female researchers are most significantly underrepresented in the field of Engineering and Technology. Despite the difference in gender ratios by discipline, all fields showed a tendency towards a closing gender gap among both all researchers and migrant researchers over time, generally in line with the overall trends across all disciplines combined (black lines).

Given the differences in gender ratios across fields of study, and to adjust for any compositional differences in the size of fields over time, we standardized the gender ratios by using a fixed (standardized) distribution of researcher populations by field as the weight. More specifically, we applied the field distribution of global researchers at the first period to the field-specific gender ratios of researchers at each period to obtain the (field-) standardized gender ratios. The results before and after standardization based on the fields of speciality showed consistent trends (see Fig. S10). This analysis shows that changes in the composition of disciplines over time does not drive the trend, i.e. observed changes in the gender gap in scholarly mobility over time that we present in the main text.

**Imputation for missing gender.** In our gender identification for authors using their first names (as described above), we had 10.73% of authors labelled with "unknown gender". To assess the robustness of our results, we further examined the impact of these researchers without given genders and to assess the sensitivity of our results to missing names. We imputed the

missing genders for the researchers with "unknown gender" using Multiple Imputation by Chained Equations (MICE), which is a widely-used method for dealing with missing data (14). More specifically, we selected the researchers' fields of research (e.g., Agricultural Sciences, Engineering and Technology, Humanities, Medical and Health Sciences, Natural Sciences or Social Sciences, the assignment method is discussed above) and time periods of their first publications (1, 2, 3, or 4 for 1998–2002, 2003–2007, 2008–2012, and 2013–2017, respectively) as features to predict the gender (female or male) of researchers with unknown gender. Instead of randomly assigning genders to these researchers, we aimed to see how gender ratios change given that we have relatively reasonable gender information imputed for these researchers. The original global-level gender ratios without "unknown gender" researchers and the result involving these researchers after assigning imputed genders are compared in Fig. S11.

For migrant researchers (orange lines), the results show little difference before and after imputing missing genders. While for the population of all researchers the gaps became wider at some time periods, the overall trends kept consistent over time.

In Fig. 1 of the main text, we used the gaps between the 45 degree lines and the fitted regression lines, which model the relationship between the gender ratios of migrant researchers and those of all researchers, to track the convergence tendency of female representation in the group of mobile researchers versus that in the total researcher population. The gaps at the X value of one in each time period are 0.42, 0.34, 0.28, and 0.24 (see Fig. 1 in the main text). After incorporating the researchers with imputed gender, the corresponding gaps change to the values of 0.45, 0.34, 0.30, and 0.25 and the country-level gender ratios of both researcher populations can be seen in Fig. S12. The gaps throughout the four periods kept consistent, and it is reasonable to believe our findings are not skewed by the composition of those researchers for whom we cannot infer gender by names.

**Table S1. Two-year padding for the residence countries between publishing years**

| Case | Year gap | Filling instance |
|---|---|---|
| 1 | $(T_{i+1} - T_i) = 2$ | $(T_{i+1} - 1, C_{i+1})$ |
| 2 | $(T_{i+1} - T_i) = 3$ | $(T_i + 1, C_i), (T_{i+1} - 1, C_{i+1})$ |
| 3 | $(T_{i+1} - T_i) = 4$ | $(T_i + 1, C_i), (T_{i+1} - 2, C_{i+1}),$ $(T_{i+1} - 1, C_{i+1})$ |
| 4 | $(T_{i+1} - T_i) \leqslant 5$ | $(T_i + 1, C_i), (T_i + 2, C_i),$ $(T_{i+1} - 2, C_{i+1}), (T_{i+1} - 1, C_{i+1})$ |

**Table S2. Validation results of the name-based gender inference process**

| Country/region | Precision rate | | Recall rate | |
|---|---|---|---|---|
| | **Female** | **Male** | **Female** | **Male** |
| Arabic/ Persian | 94.91% | 95.39% | 96.96% | 92.89% |
| Chinese | 50.55% | 87.60% | 50.30% | 50.05% |
| German | 98.35% | 97.54% | 98.06% | 98.00% |
| Japanese | 90.49% | 91.26% | 92.34% | 98.72% |
| Indian | 90.49% | 80.92% | 96.25% | 96.22% |
| Russian | 97.73% | 96.83% | 99.23% | 97.75% |

**Table S3. The stock and proportion of migrant researchers by gender.** In each period, the countries are sorted based on the number of female researchers in a descending order. The last row is the number of all migrant researchers by gender and the respective proportions of researchers across the globe.

| | Period 1 (1998-2002) | | | |
|---|---|---|---|---|
| | Female migrants | | Male migrants | |
| Country | Stock | Proportion (%) | Stock | Proportion (%) |
| United States | 10,830 | 4.95 | 33,808 | 7.33 |
| United Kingdom | 4,464 | 8.74 | 12,475 | 11.53 |
| Germany | 2,645 | 8.80 | 9,721 | 10.54 |
| Canada | 2,345 | 8.62 | 6,815 | 12.91 |
| France | 2,312 | 8.16 | 6,334 | 11.00 |
| Italy | 1,719 | 4.94 | 3,530 | 7.26 |
| Australia | 1,454 | 7.71 | 4,338 | 12.31 |
| Japan | 1,289 | 3.34 | 7,414 | 3.79 |
| Spain | 1,231 | 4.86 | 2,646 | 6.27 |
| China | 1,223 | 3.07 | 3,863 | 3.50 |
| Switzerland | 1,136 | 16.76 | 3,785 | 19.05 |
| Netherlands | 994 | 9.37 | 3,339 | 12.19 |
| Sweden | 878 | 8.17 | 2,442 | 11.74 |
| Brazil | 652 | 3.33 | 1,748 | 6.43 |
| South Korea | 522 | 5.33 | 2,152 | 7.30 |
| Israel | 502 | 7.38 | 1,622 | 11.71 |
| India | 456 | 3.52 | 1,503 | 5.42 |
| Global | 29,161 | 4.28 | 91,684 | 5.96 |

| | Period 2 (2003-2007) | | | |
|---|---|---|---|---|
| | Female migrants | | Male migrants | |
| Country | Stock | Proportion (%) | Stock | Proportion (%) |
| United States | 13,585 | 4.52 | 35,308 | 6.52 |
| United Kingdom | 5,999 | 8.49 | 13,809 | 10.76 |
| Canada | 3,428 | 8.54 | 8,440 | 12.90 |
| Germany | 3,348 | 7.45 | 10,235 | 9.26 |
| France | 3,110 | 8.29 | 7,076 | 10.44 |
| Australia | 2,297 | 7.96 | 5,350 | 12.11 |
| China | 2,230 | 2.19 | 6,379 | 2.41 |
| Italy | 2,009 | 4.28 | 3,538 | 6.15 |
| Spain | 1,676 | 4.33 | 3,103 | 5.50 |
| Switzerland | 1,614 | 15.95 | 4,475 | 18.45 |
| Japan | 1,511 | 2.93 | 7,312 | 3.30 |
| Netherlands | 1,237 | 7.96 | 3,079 | 9.66 |
| Sweden | 1,018 | 7.33 | 2,336 | 10.32 |
| South Korea | 936 | 4.70 | 3,168 | 6.24 |
| Brazil | 882 | 2.11 | 1,727 | 3.62 |
| India | 726 | 3.36 | 2,269 | 5.07 |
| Israel | 457 | 5.49 | 1,284 | 8.42 |
| Global | 41,717 | 3.96 | 108,958 | 5.28 |

| | Period 3 (2008-2012) | | | |
|---|---|---|---|---|
| | Female migrants | | Male migrants | |
| Country | Stock | Proportion (%) | Stock | Proportion (%) |
| United States | 17,659 | 4.52 | 39,872 | 6.37 |
| United Kingdom | 8,295 | 9.11 | 16,179 | 10.96 |
| Germany | 5,050 | 8.11 | 12,620 | 10.10 |
| Canada | 4,621 | 8.43 | 9,833 | 12.32 |
| France | 4,389 | 9.02 | 8,857 | 11.26 |
| China | 4,315 | 2.05 | 10,974 | 2.15 |
| Australia | 3,827 | 9.19 | 7,490 | 13.12 |
| Spain | 2,786 | 5.12 | 4,542 | 6.36 |
| Switzerland | 2,675 | 17.86 | 6,558 | 21.78 |
| Italy | 2,460 | 4.23 | 4,025 | 6.23 |
| Netherlands | 1,961 | 8.80 | 4,196 | 11.28 |
| Japan | 1,912 | 3.07 | 7,450 | 3.17 |
| Sweden | 1,609 | 9.50 | 3,035 | 12.18 |
| South Korea | 1,362 | 3.79 | 4,548 | 5.63 |
| India | 1,326 | 3.38 | 4,062 | 5.33 |
| Brazil | 1,229 | 1.65 | 2,294 | 3.12 |
| Israel | 536 | 5.71 | 1,409 | 8.92 |
| Global | 61,699 | 4.03 | 140,594 | 5.18 |

| | Period 4 (2013-2017) | | | |
|---|---|---|---|---|
| | Female migrants | | Male migrants | |
| Country | Stock | Proportion (%) | Stock | Proportion (%) |
| United States | 20,600 | 5.19 | 43,242 | 7.01 |
| United Kingdom | 10,456 | 11.13 | 18,615 | 12.70 |
| China | 6,969 | 2.24 | 18,107 | 2.53 |
| Germany | 6,290 | 10.29 | 13,764 | 11.66 |
| Canada | 5,437 | 9.65 | 10,111 | 12.80 |
| France | 5,351 | 10.44 | 9,914 | 12.54 |
| Australia | 4,879 | 10.58 | 8,784 | 13.99 |
| Switzerland | 3,454 | 20.63 | 6,896 | 22.17 |
| Spain | 3,192 | 5.58 | 4,629 | 6.35 |
| Italy | 3,085 | 5.21 | 4,910 | 7.73 |
| Netherlands | 2,619 | 10.85 | 4,585 | 12.36 |
| Sweden | 2,161 | 12.70 | 3,832 | 15.28 |
| Japan | 2,011 | 3.59 | 7,162 | 3.42 |
| India | 2,001 | 3.98 | 5,639 | 5.98 |
| Brazil | 1,785 | 2.18 | 3,073 | 3.81 |
| South Korea | 1,762 | 4.12 | 5,137 | 5.57 |
| Israel | 674 | 7.68 | 1,642 | 10.97 |
| Global | 79,370 | 4.65 | 167,182 | 5.66 |

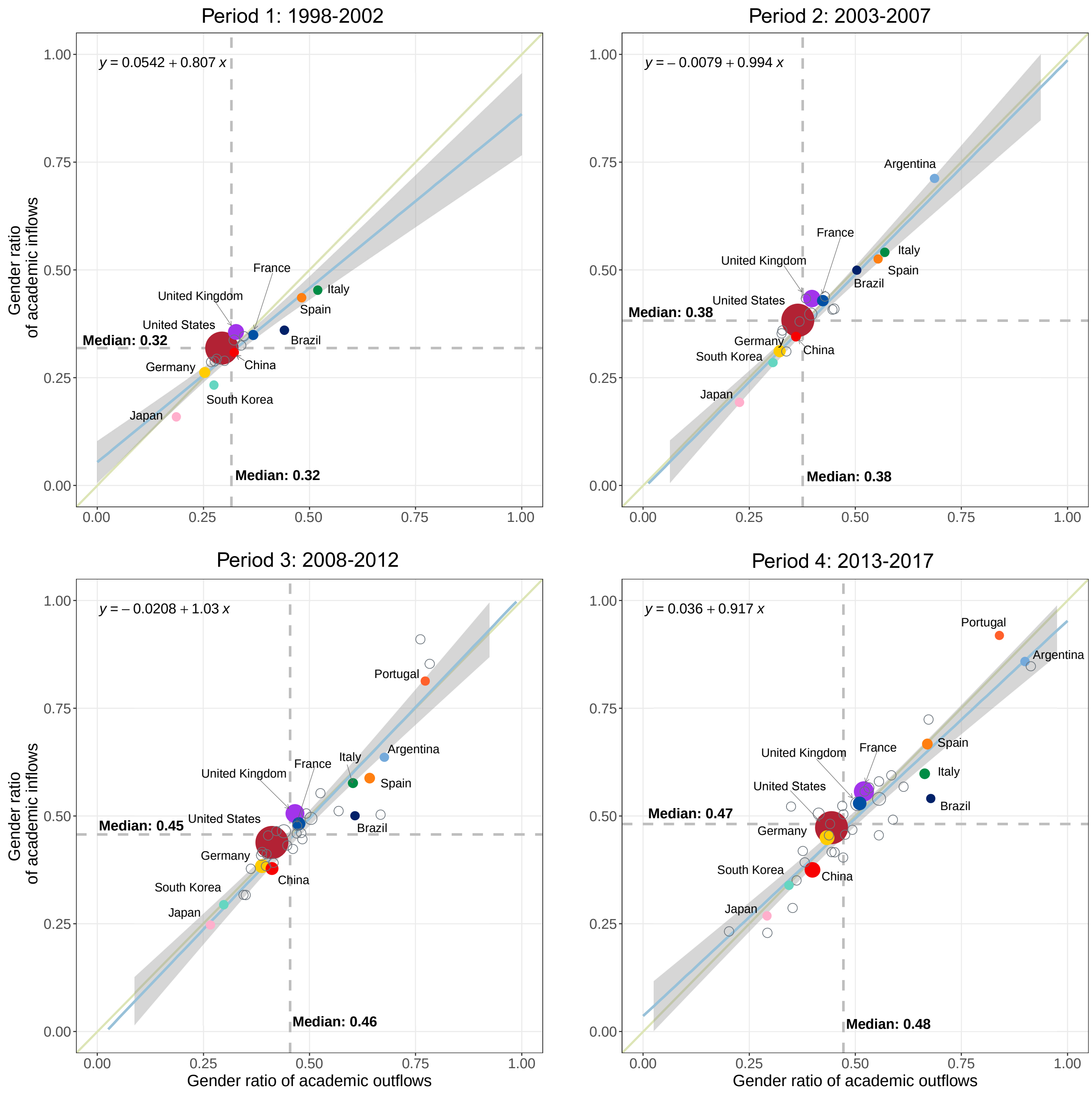


**Fig. S1.** The comparison of gender ratios among scholarly outflows (X-axis) and inflows (Y-axis) during the four periods

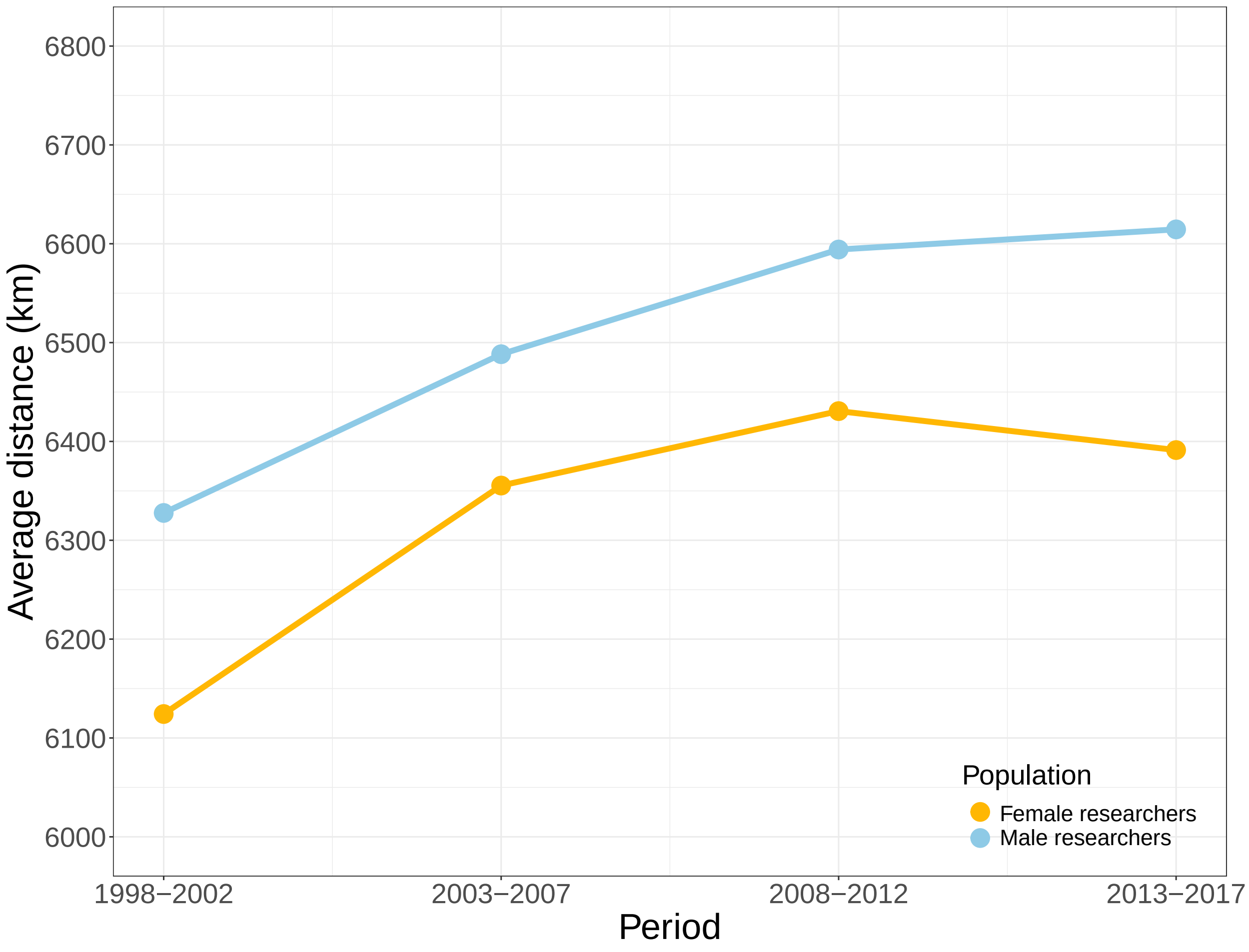


**Fig. S2.** Average distance between origin and destination of migration among female and male researchers

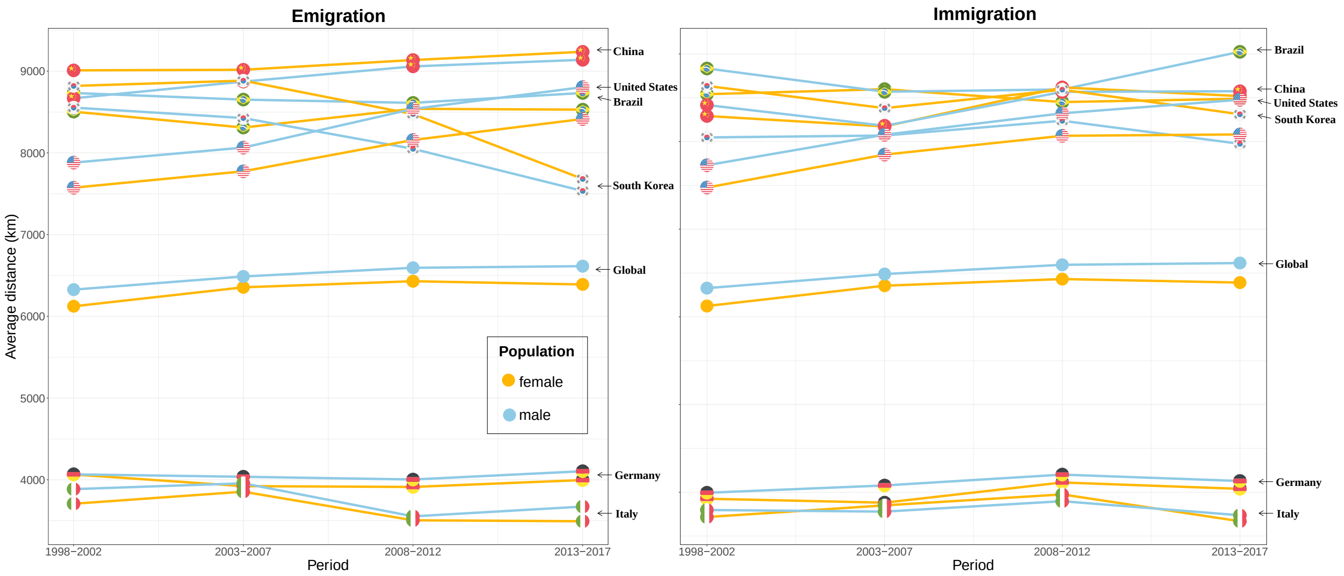


**Fig. S3.** Average distance among female and male emigrant (left) and immigrant (right) researchers across selected countries (the lines without flags show the global average)

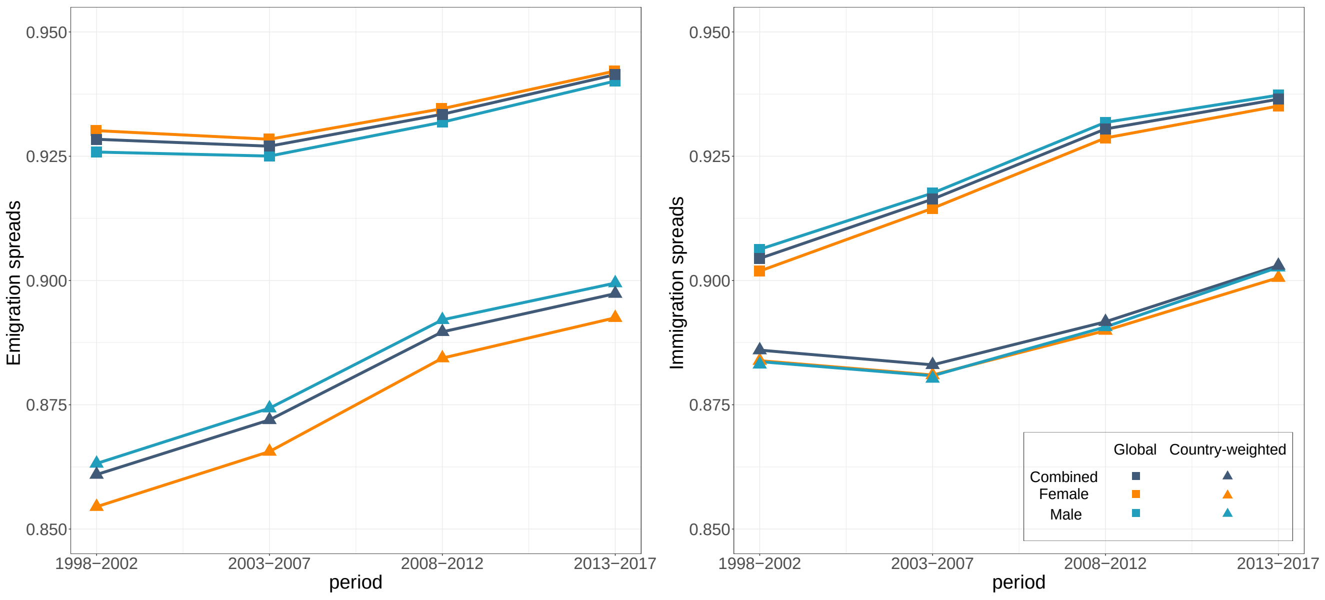


**Fig. S4.** Global-level emigration (left) and immigration (right) scholarly migration spreads by gender

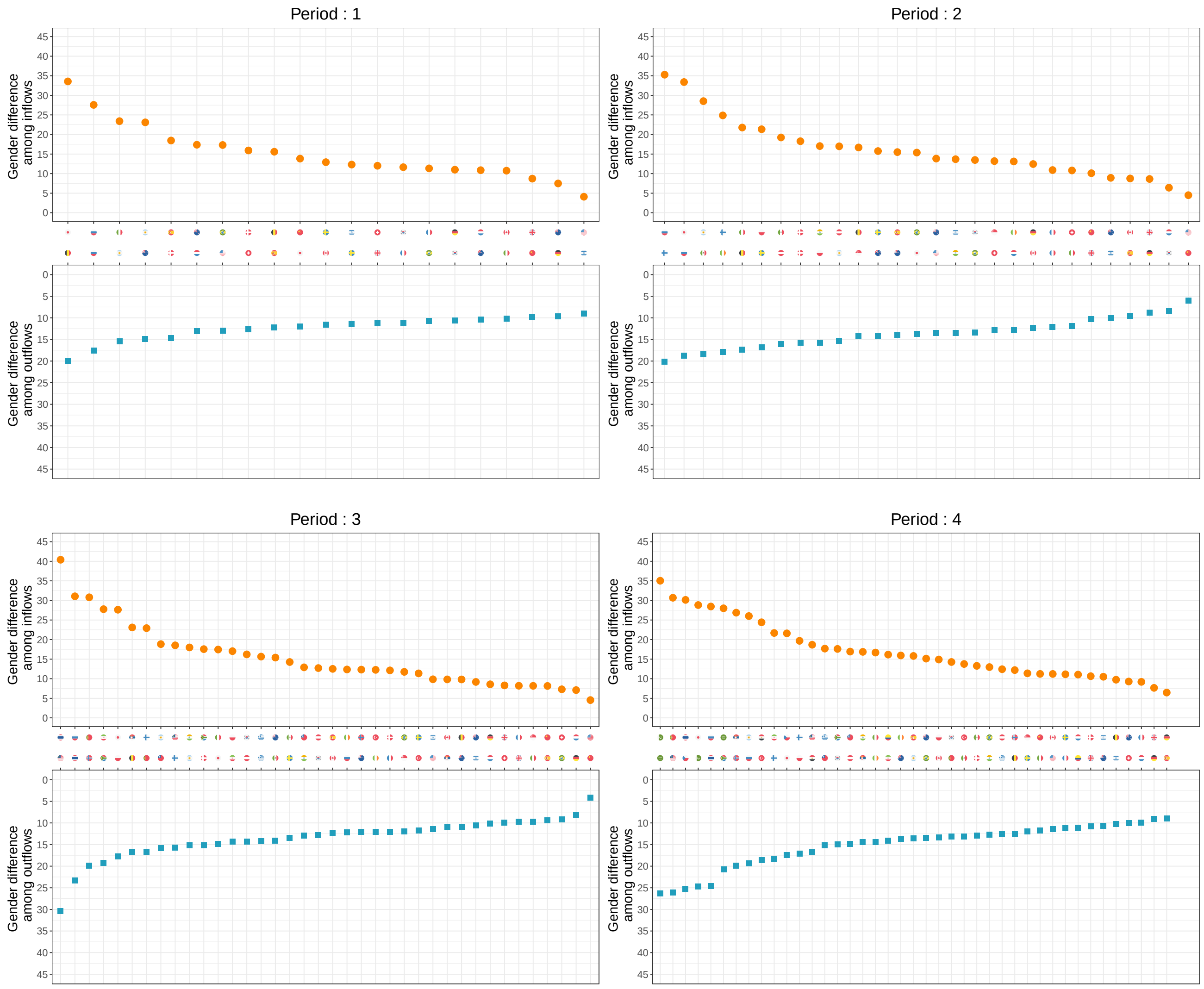


**Fig. S5.** Gender difference by destination and origin country in four periods by inflows (top part of each panel) and outflows (bottom part of each panel)

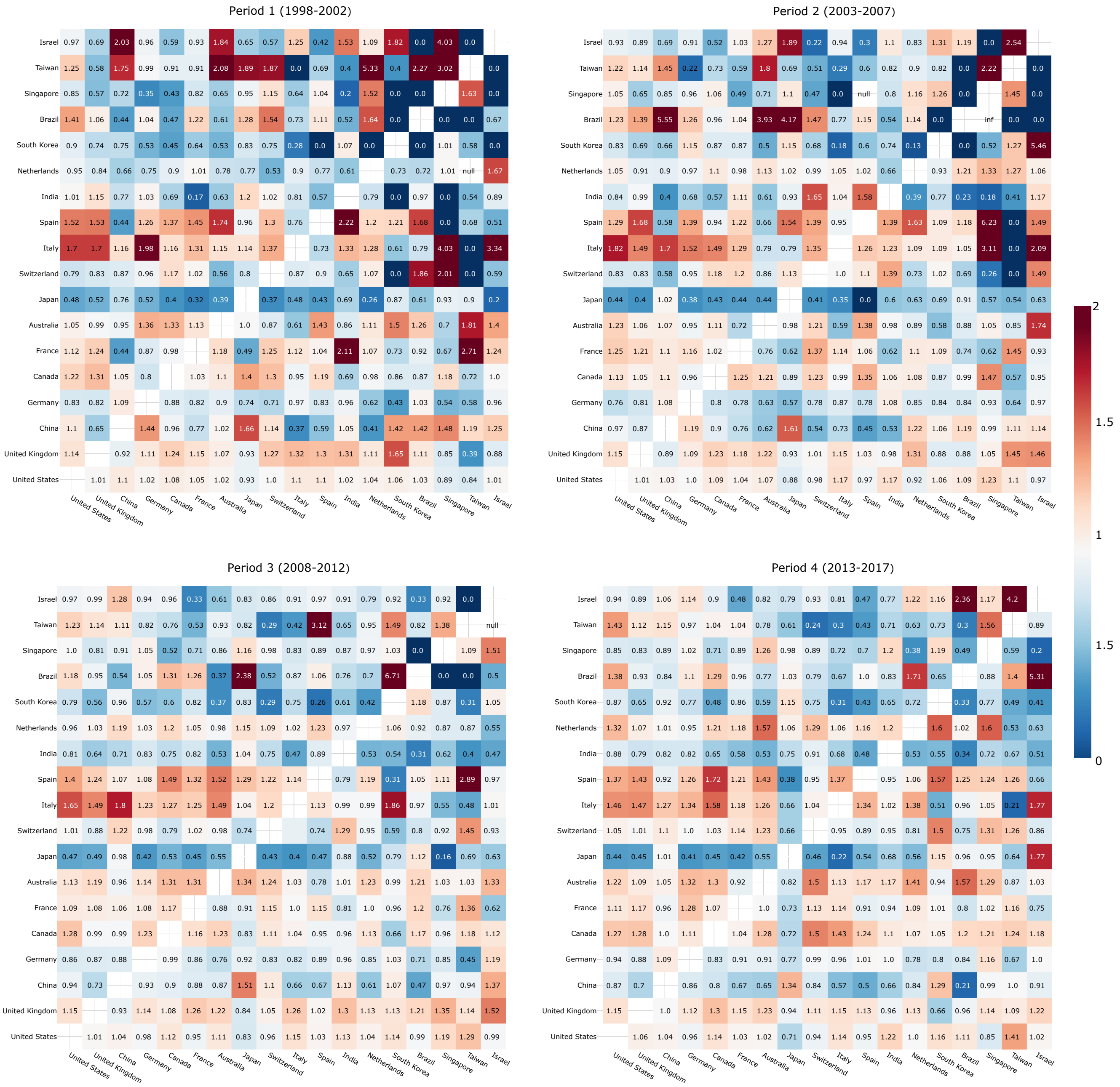


**Fig. S6.** Gender ratio among migrant researchers by scholarly migration path

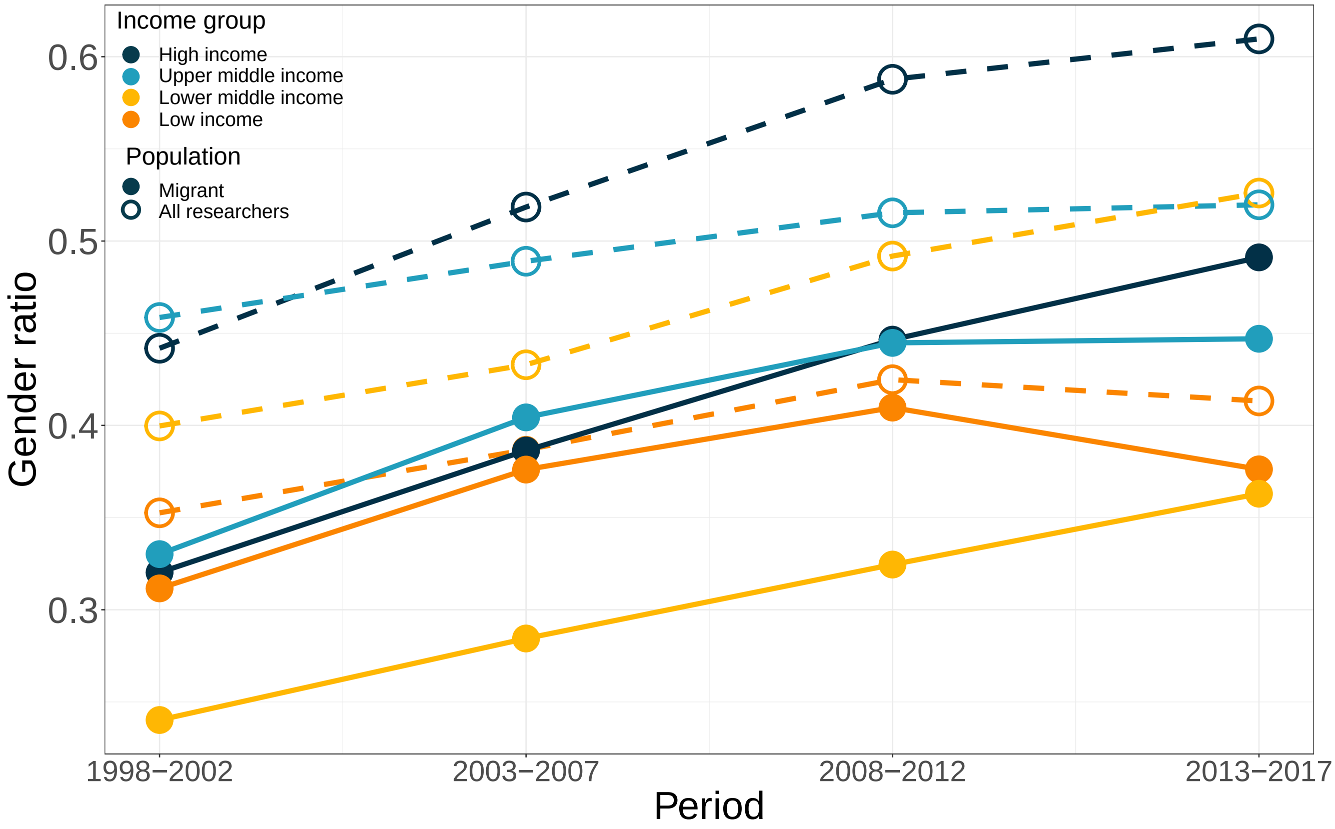


**Fig. S7.** Gender ratios among all published researchers and migrant researchers across income groups (The classification of income groups are assigned by World Bank)

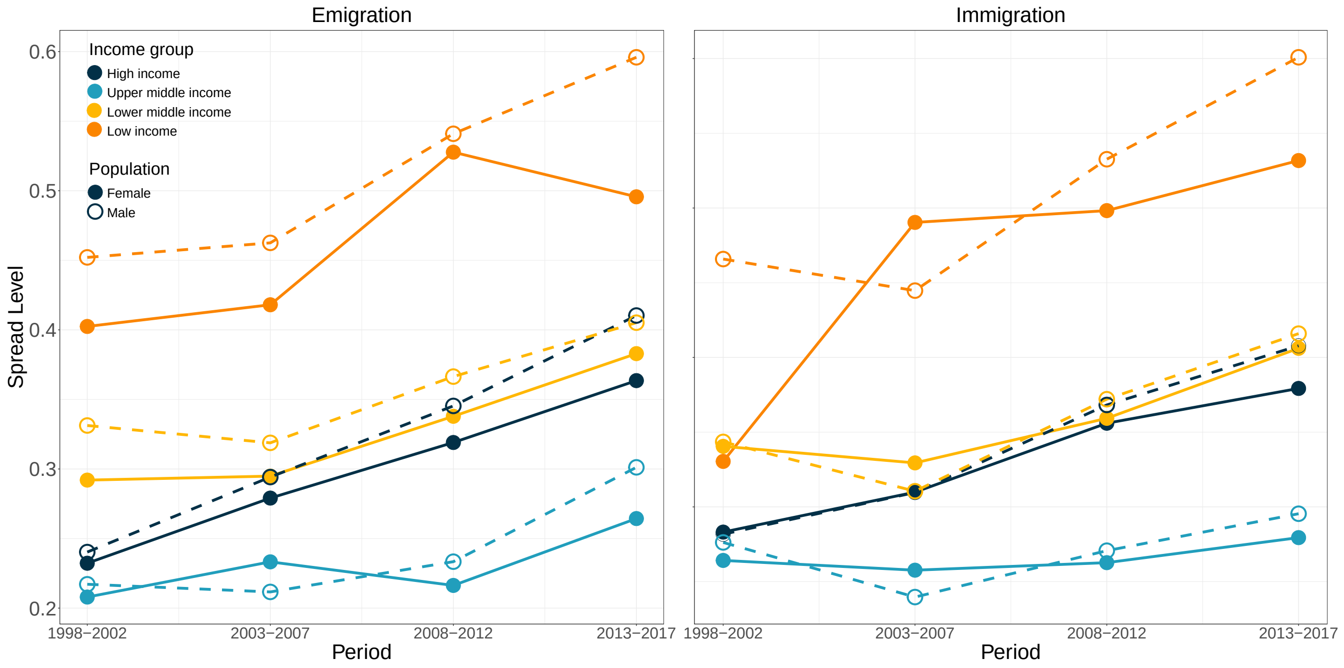


**Fig. S8.** Scholarly emigration (left) and immigration (right) spreads, by gender and across income groups

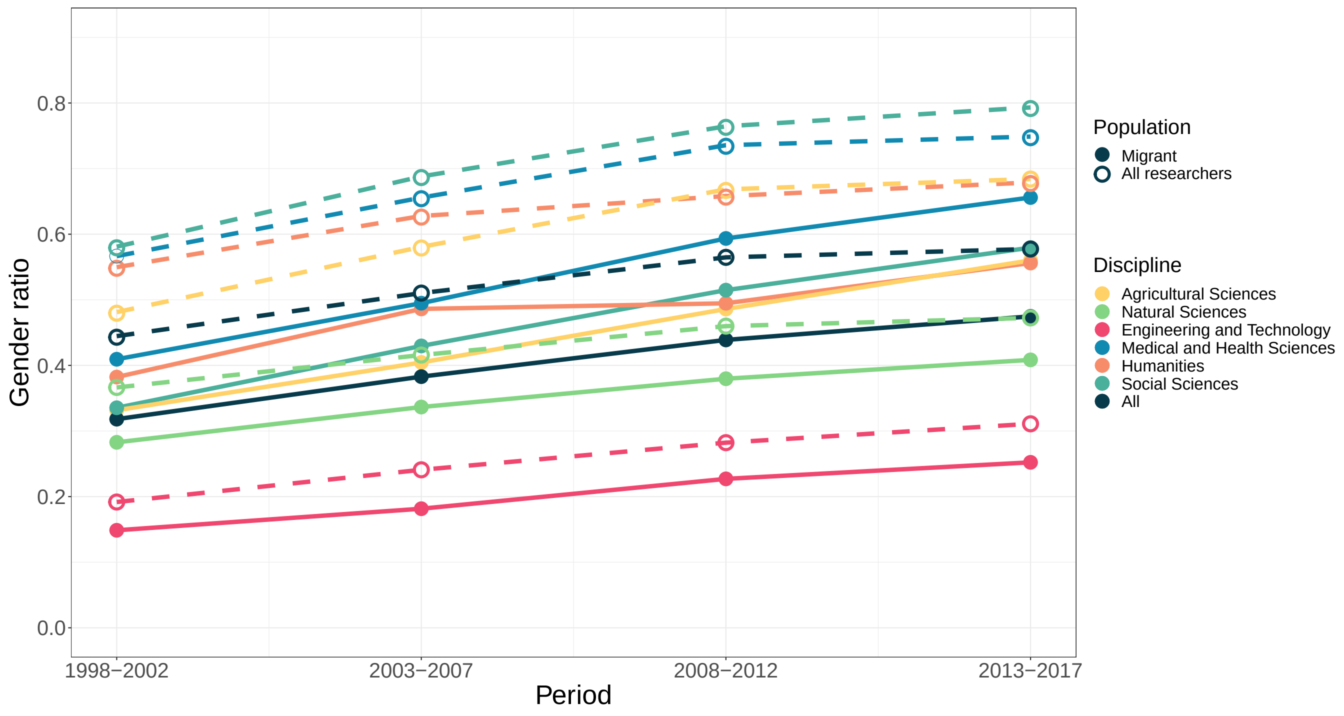


**Fig. S9.** Gender ratios among all published researchers and migrant researchers by discipline

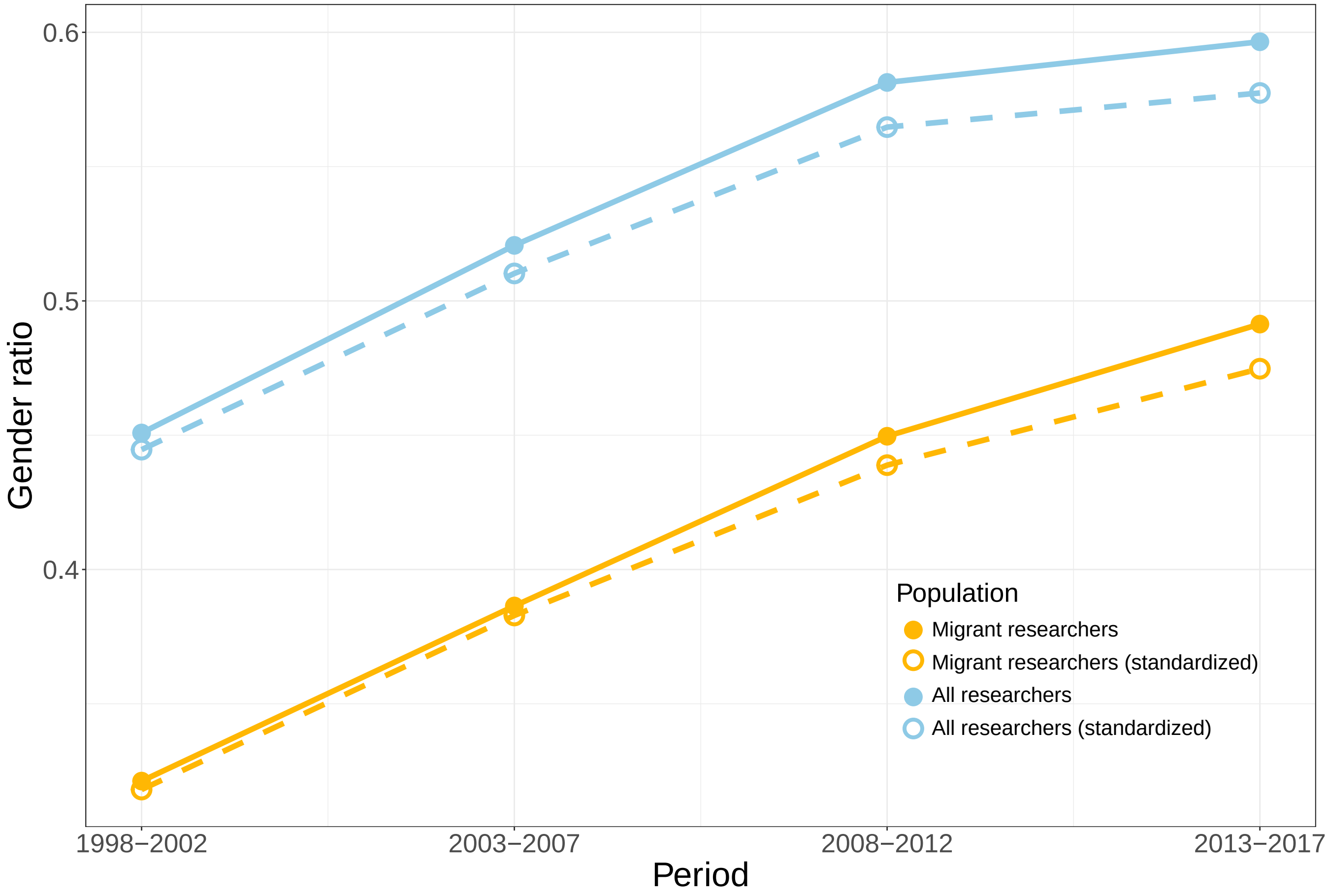


**Fig. S10.** The comparison between the gender ratios among all published researchers and migrant researchers before and after field-standardization

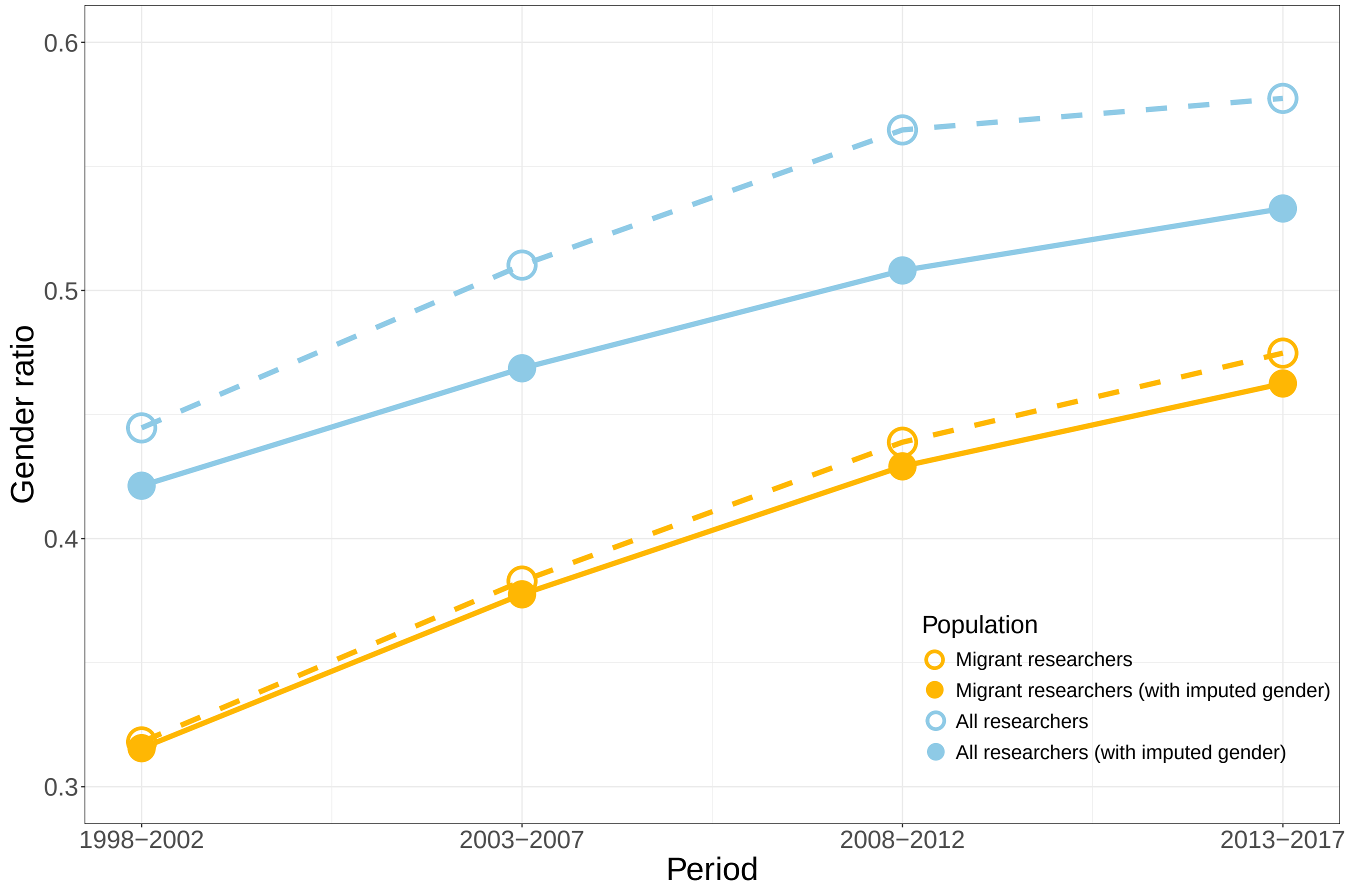


**Fig. S11.** Gender ratios among all published researchers and migrant researchers before and after imputing missing genders

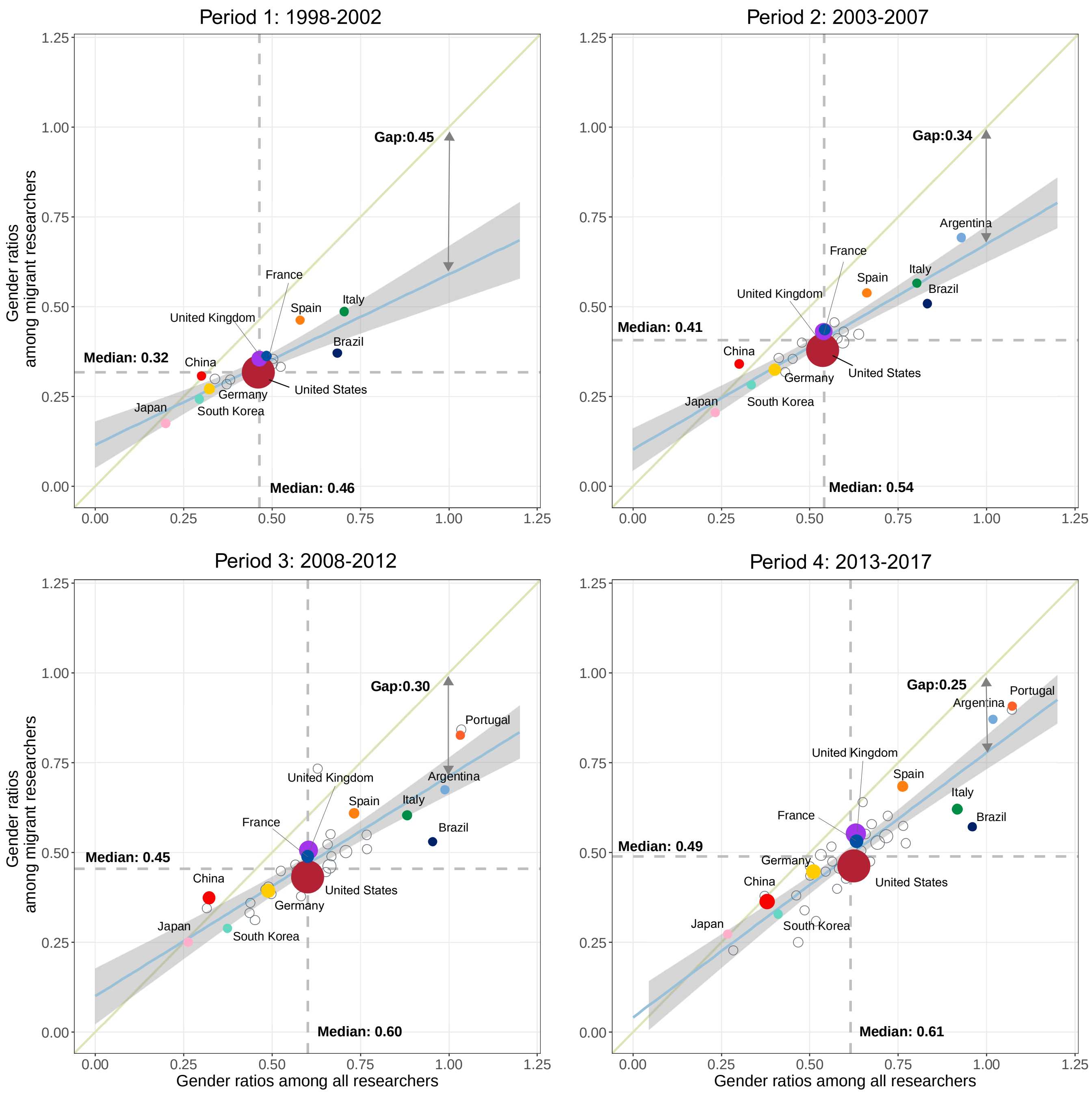


**Fig. S12.** Gender ratios among all published researchers (X-axis) and migrant researchers (Y-axis) (after imputing missing genders)

## References


1. B Björk, D Solomon, The publishing delay in scholarly peer-reviewed journals. *J. Informetrics* **7**, 914–923 (2013).
2. J Baas, M Schotten, A Plume, G Côté, R Karimi, Scopus as a curated, high-quality bibliometric data source for academic research in quantitative science studies. *Quant. Sci. Stud.* **1**, 377–386 (2020).
3. M Winkelmann, List of first names, genders and country-specific frequencies (2022) original-date: 2016-09-08T01:46:31Z.
4. A Akbaritabar, F Squazzoni, Gender patterns of publication in top sociological journals. *Sci. Technol. & Hum. Values* **46**, 555–576 (2021).
5. D Kozlowski, V Larivióre, CR Sugimoto, T Monroe-White, Intersectional inequalities in science. *Proc. Natl. Acad. Sci.* **119**, 2 (2022).
6. X Zhao, S Aref, E Zagheni, G Stecklov, International migration in academia and citation performance: An analysis of german-affiliated researchers by gender and discipline using scopus publications 1996-2020 in *Proceedings of the 18th International Conference on Scientometrics and Informetrics (ISSI 2021).* (The 18th International Conference on Scientometrics and Informetrics (ISSI), (2021).
7. X Zhao, S Aref, E Zagheni, G Stecklov, Return migration of german-affiliated researchers: Analyzing departure and return by gender, cohort, and discipline using scopus bibliometric data 1996-2020. *Scientometrics* **127** (2022).
8. V Macháček, M Srholec, MR Ferreira, N Robinson-Garcia, R Costas, Researcher's institutional mobility: bibliometric evidence on academic inbreeding and internationalization. *Sci. Public Policy* **49**, 85–97 (2022).
9. M Czaika, H de Haas, The globalization of migration: Has the world become more migratory? *Int. Migr. Rev.* **48**, 283–323 (2014).
10. World bank country and lending groups (https://datahelpdesk.worldbank.org/knowledgebase/articles/906519-world-bank-country-and-lending-groups/) (2021).
11. A Bailey, C Mulder, Highly skilled migration between the global north and south: gender, life courses and institutions. *J. Ethn. Migr. Stud.* **43**, 2689–2703 (2017).
12. K Donato, D Gabaccia, *Gender and International Migration: From the Slavery Era to the Global Age.* (Russell Sage Foundation), (2015).
13. Organisation for Economic Co-operation and Development (OECD), Revised field of science and technology (fos) classification in the frascati manual (classification, field of science and technology classification, fos, frascati, methodology, research and development), Technical report (2007).
14. M Azur, E Stuart, C Frangakis, P Leaf, Multiple imputation by chained equations: what is it and how does it work? *Int. J. Methods Psychiatr. Res.* **20**, 40–49 (2011).
